# Clade F AAVHSCs cross the blood brain barrier and transduce the central nervous system in addition to peripheral tissues following intravenous administration in nonhuman primates

**DOI:** 10.1371/journal.pone.0225582

**Published:** 2019-11-26

**Authors:** Jeff L. Ellsworth, Jacinthe Gingras, Laura J. Smith, Hillard Rubin, Tania A. Seabrook, Kruti Patel, Nicole Zapata, Kevin Olivieri, Michael O’Callaghan, Elizabeth Chlipala, Pablo Morales, Albert Seymour

**Affiliations:** 1 Homology Medicines, Inc., Bedford, Massachusetts, United States of America; 2 Premier Laboratory, LLC., Boulder, Colorado, United States of America; 3 Mannheimer Foundation, Inc., Homestead, Florida, United States of America; PTC Therapeutics, UNITED STATES

## Abstract

The biodistribution of AAVHSC7, AAVHSC15, and AAVHSC17 following systemic delivery was assessed in cynomolgus macaques (*Macaca fascicularis*). Animals received a single intravenous (IV) injection of a self-complementary AAVHSC-enhanced green fluorescent protein (eGFP) vector and tissues were harvested at two weeks post-dose for anti-eGFP immunohistochemistry and vector genome analyses. IV delivery of AAVHSC vectors produced widespread distribution of eGFP staining in glial cells throughout the central nervous system, with the highest levels seen in the pons and lateral geniculate nuclei (LGN). eGFP-positive neurons were also observed throughout the central and peripheral nervous systems for all three AAVHSC vectors including brain, spinal cord, and dorsal root ganglia (DRG) with staining evident in neuronal cell bodies, axons and dendritic arborizations. Co-labeling of sections from brain, spinal cord, and DRG with anti-eGFP antibodies and cell-specific markers confirmed eGFP-staining in neurons and glia, including protoplasmic and fibrous astrocytes and oligodendrocytes. For all capsids tested, 50 to 70% of glial cells (S100-β+) and on average 8% of neurons (NeuroTrace+) in the LGN were positive for eGFP expression. In the DRG, 45 to 62% of neurons and 8 to 12% of satellite cells were eGFP-positive for the capsids tested. eGFP staining was also observed in peripheral tissues with abundant staining in hepatocytes, skeletal- and cardio-myocytes and in acinar cells of the pancreas. Biodistribution of AAVHSC vector genomes in the central and peripheral organs generally correlated with eGFP staining and were highest in the liver for all AAVHSC vectors tested. These data demonstrate that AAVHSCs have broad tissue tropism and cross the blood-nerve and blood-brain-barriers following systemic delivery in nonhuman primates, making them suitable gene editing or gene transfer vectors for therapeutic application in human genetic diseases.

## Introduction

The introduction of adeno-associated viruses (AAVs) as vectors for transgene delivery has had a significant impact on clinical development of genetic therapies for a range of human diseases [1–8]. Driving the interest in using AAVs for gene therapy (GT) are several well-described attributes of this group of parvoviruses: their ability to transduce both dividing and non-dividing cells, their relatively low toxicity and immunogenicity and their wide tissue tropism. Improved manufacturing methods for scalable, high-yield production of recombinant AAVs and enhanced screening and isolation of AAV capsids with novel characteristics have further enabled development of AAVs for GT [9].

A group of fifteen AAVs isolated from normal human CD34+ hematopoietic stem cells, termed AAVHSCs, have been described[10]. More recently, we have shown that the AAVHSCs can mediate nuclease-free gene editing with high efficiencies [11] and can also be used for gene transfer using transgenes under the control of exogenous promoters [12, 13]. Analysis of the AAVHSC capsid sequences revealed that these AAVHSCs all fall within AAV Clade F [14]. A property of Clade F vectors is the ability to traverse the blood-brain-barrier (BBB) following intravascular administration, resulting in transduction of neurons and glia in neonatal and adult mice [15, 16], rats [17], cats [16] and in nonhuman primates [18, 19]. These data suggested that systemic delivery of a BBB-penetrating AAV could be used to deliver therapeutic genes to the central nervous system (CNS). Extended to human patients with spinal muscular atrophy, systemic delivery of AAV9-*SMN1* increased survival, improved motor milestone achievement and partially restored motor function [5]. These data led to the recent approval of Zolgensma® for pediatric patients with spinal muscular atrophy [20].

As we are developing the AAVHSCs for use as gene editing and gene therapy vectors for treatment of rare genetic diseases in humans, it was necessary to characterize their biodistribution in nonhuman primates. Cynomolgus macaques were used in the present work for their close phylogenetic relationship to humans and to avoid disparities in CNS biodistribution previously reported between mice and nonhuman primates [21]. Data gained from such studies provide not only the tissue and associated cell type tropism of the AAVHSCs but also inform selection of potential therapeutic indication(s) for these vectors.

To evaluate the ability of AAVHSCs in crossing the blood-nerve-barrier (BNB) and BBB after systemic delivery and assess their cellular tropism in the CNS and peripheral organs, AAVHSC7, AAVHSC15 and AAVHSC17 were studied in four to five month-old cynomolgus macaques, an age where the permeability of the BBB is considered mature [22, 23]. These vectors were chosen based on tissue tropism observed in mice where AAVHSC7, AAVHSC13, AAVHSC15 and AAVHSC17 showed extensive liver tropism and wide tissue distributions [24]. Only AAVHSC7, AAVHSC15 and AAVHSC17 were pursued in nonhuman primate studies due to AAVHSC13 and AAVHSC17 having the same amino acid sequence [10]. These three AAVHSC capsids showed high packaging efficiencies and could be produced in amounts sufficient for biodistribution studies in large animals facilitating their use in the present study in nonhuman primates. Each vector packaged a self-complementary (sc) eGFP reporter transgene driven by a ubiquitously-expressing promoter and was administered to cynomolgus macaques by a single IV infusion. Our data show a widespread rostro-caudal transduction gradient of neurons and glia within the CNS by the AAVHSCs including most regions of the brain and spinal cord. High-level transduction of the dorsal root ganglia (DRG) and other peripheral tissues was also observed with eGFP expression highest in liver for all three AAVHSCs tested. Extensive eGFP immunostaining was also observed in skeletal- and cardio-myocytes, pancreas, and in the kidneys.

## Materials and methods

### Animal care and use

Housing and all procedures were in accordance to the Institutional Animal Care and Use Committee at the Mannheimer Foundation (Homestead, FL). All breeding, housing, and procedures were performed on cynomolgus macaques (*Macaca fascicularis*) at the Mannheimer Foundation, Inc. under Protocol Number 2016–05. All procedures were performed under ketamine anesthesia and all efforts were made to minimize suffering. Social housing in large outdoor pens is the primary housing method for all colony animals. No animal is isolated in areas without other animals. Animals greater than 6 months of age may be temporarily housed in single cages for clinical reasons (treatments). Animal may also be housed in single cages when the IACUC approves it for research that provides adequate scientific justification. All animals that must be temporarily housed singly, are provided with visual, auditory, and olfactory contact with other animals. Further, connecting cages are used whenever possible to allow tactile contact or pair housing. All cages are equipped with mirrors to allow self-viewing and as an aid to view room activities. Positive human contact is also added as it has been shown to reduce abnormal behavior activities as well as anxiety and fear. Additional enrichment may be provided during single housing and may include the use of puzzle feeders and/or other feeding enrichments to insure/encourage species typical foraging activities.

All singly-housed animals are fed at least once daily in bowls or feed cups mounted on the individual cages. A small portion is offered to those cages where no food is present early in the morning–and/or those that will be cleaned later on that day. Group housed animals are fed daily using galvanized type or polypropylene feeders. Purina LabDiet products are used at the Mannheimer Foundation facilities (LabDiet 5049) Fruits, vegetables are other healthy food items approved by the attending veterinarian, are offered on a daily basis to both indoor and outdoor housed animals.

Anesthesia and analgesia guidelines are provided, based on current literature and veterinarian recommendations. Veterinarians are consulted by researchers about the selection and use of anesthetics/analgesics, as required by the Mannheimer Foundation IACUC, which follows a veterinary consultation process prior to the use of analgesics, anesthesia, sedation, tranquilization and euthanasia. The Mannheimer Foundation veterinary staff directly administer anesthetics, and provides post-operative care, including analgesia. Animal health records are closely monitored by the veterinary staff to verify that anesthetics and analgesics are being administered and properly recorded per protocol directions.

Euthanasia is always performed by overdose of barbiturates. Euthanasia procedures are performed by veterinarians (or veterinary assistants under supervision of veterinarians)–and according the *2013 AVMA Guidelines on Euthanasia*. Any deviation from approved procedures requires scientific justification in the form of an IACUC-approved protocol. No animals are euthanized in rooms or areas where other animals are present. The Mannheimer Foundation Institutional Animal Care and Use Committee (IACUC) specifically approved this study.

### AAVHSC vector production

All vectors were produced by triple transfection in HEK293 cells and purified through two rounds of CsCl density gradient ultracentrifugation at SABTech, Inc., (Philadelphia, PA). All AAVHSC vectors were formulated in PBS (180mM NaCl, 10mM sodium phosphate, pH7.3) with 0.001% Pluronic F68. Vector titers were determined by qPCR using primers and probes specific for eGFP.

### IV delivery of scAAVHSC-CBA-eGFP in nonhuman primates

Sera from all animals were prescreened for anti-AAVHSC neutralizing antibodies using the Huh-7 cell-based assay [Horae Gene Therapy Center, University of Massachusetts (Worcester, MA)] over a range of serum dilutions from 1/10 to 1/1250. Only antibody negative animals were used in this work. Veterinary staff at the Mannheimer Foundation anesthetized each subject (young males, 4–5 months old) with an intramuscular injection of ketamine (10 mg/kg). Each animal was fitted with a saphenous vein catheter for IV injection of AAVHSCs. Blood samples were collected from the femoral vein for assessment of clinical chemistries, CBCs, and neutralizing antibodies at baseline. AAVHSCs were infused at doses of 1.0 x 10^14^ vg/kg for scAAVHSC17-CBA-eGFP (n = 2 animals) and 0.7 x 10^14^ vg/kg for scAAVHSC15-CBA-eGFP (n = 2 animals) and scAAVHSC7-CBA-eGFP (n = 1 animal) or an equivalent volume of vehicle alone (n = 1 animal) at 7–8 mL/kg through a saphenous vein catheter over 1.0 min using all-plastic syringes. Each animal was allowed to recover in a warmed incubator following injection and, when fully awake was returned to the cage with its mother. At two weeks post-dosing, all subjects were anesthetized with ketamine, heparinized (5,000 units, given 5–10 min prior to sacrifice) and euthanized with an overdose of IV sodium pentobarbital (80–100 mg/kg).

### Perfusion and tissue processing

Immediately following euthanasia, each animal was trans-cardially perfused with 1.0L of phosphate-buffered saline followed by 1.0L of 4% paraformaldehyde (PFA) in phosphate-buffered saline (PBS), pH7.4 (Polyscientific R & D, Bay Shore, NY). Tissues were excised and were post-fixed in fresh 4% PFA in PBS, pH7.4 for 48-h at room temperature and were then transferred to PBS + 0.05% sodium azide (Teknova, Hollister, CA) and stored at 4° C.

### Histology and microscopy

Non-CNS paraffin-embedded tissues (except dorsal root ganglia) were sectioned at 4–5 microns and were processed for eGFP immunohistochemistry (IHC) at Premier Laboratory (Boulder, CO). eGFP-positive cells were stained by IHC with a rabbit anti-eGFP polyclonal antibody (Abcam #ab290). Endogenous peroxidase was inhibited by incubation in 3% H_2_O_2._ An enzyme pretreatment of proteinase K was utilized, followed by a serum-free protein block. Primary antibody incubation for 30 minutes at room temperature, followed by an EnVision+ Rabbit HRP detection system. eGFP was visualized with an EnVision+Rabbit HRP kit using diaminobenzidine to stain for eGFP and hematoxylin as counterstain. The IHC slides were assessed according to the following methodology. Slides were viewed with a Nikon Eclipse 80i microscope with an attached Nikon DXM 1200C digital camera. For each IHC tissue the entire tissue was assessed, and then using Nikon Elements version 3.0 software, the approximate percentage of the tissue that was positive for the DAB signal was batched into 3 separate intensity grades: +1 (approx. 201–220 intensity signal in the software); +2 (approx. 151–200 intensity signal in the software); +3 (approx. 100–150 intensity signal in the software) A scoring example for eGFP staining in the liver is shown in S2 Fig.

CNS tissues (brains and spinal cords) and peripheral nervous system (PNS) tissues (DRG) were processed for eGFP IHC at NeuroScience Associates (Knoxville, TN). Tissues were treated with 20% glycerol and 2% dimethylsulfoxide to prevent freeze-artifacts and multiply embedded in a gelatin matrix using MultiBrain^™^ Technology (NeuroScience Associates). After curing, each block was rapidly frozen by immersion in isopentane chilled to -70°C with crushed dry ice and were mounted on a freezing stage of an AO 860 sliding microtome. The MultiBrain^™^ block was sectioned coronally at 40 μm. Approximately 1650 sections were taken per brain (macaque brains at this age are ~65 mm in length) with every 24^th^ section stained with a 960 μm interval between stained sections. Both sagittal and longitudinal sections (40 μm) of spinal cords and 40 μm sections were taken of DRG. All sections cut (none were discarded) were collected sequentially into 24 containers which were filled with Antigen Preserve solution (50% PBS pH 7.0, 50% Ethylene glycol, 1% Polyvinyl Pyrrolidone). CNS tissue sections were stained free-floating. All incubation solutions from the blocking serum onward used Tris buffered saline (TBS) with Triton X-100 (TX) as the vehicle; all rinses were with TBS. After treatment with hydrogen peroxide and blocking serum, the sections were immunostained with a GFP Tag Antibody, ABfinity^™^ Rabbit Monoclonal (Thermo Fisher Scientific, catalog # G10362, RRID AB_2536526) at a 1:500 dilution overnight at room temperature. Vehicle solution contained Triton X-100 for permeabilization. Slides were rinsed and a biotinylated goat anti-rabbit HRP secondary antibody was applied and eGFP was visualized with diaminobenzidine and counterstained with thionine light. IHC images were acquired using Aperio-Imagescope (Leica Biosystems) and Huron Viewer (Huron Digital Pathology) softwares. All eGFP positive and negative control tissues showed the expected level of staining.

All CNS, PNS and non-CNS tissue slides were read and scored for eGFP in a blinded manner by board-certified veterinary pathologists at ToxPathSpecialists (Frederick, MD) and Charter Preclinical Services (Hudson, MA), respectively.

### Immunofluorescence

A subset of brain, spinal cord, and DRG sections (40 μm) from the MultiBrain^™^ blocks were processed using immunofluorescence to co-label for eGFP and glial and/or neuronal specific markers. Free-floating sections from PFA perfused animals (see details above) were rinsed in TBS and subsequently blocked for 2.0h at room temperature with a buffer containing 5% normal donkey serum (Novus Bio catalog # NBP1-50088) and 0.25% Triton X-100 (ThermoFisher Scientific catalog # 85111) in TBS. Sections were incubated in primary antibodies diluted in blocking buffer overnight at 4°C. eGFP signal was boosted by using a chicken polyclonal anti-eGFP (Aves Labs catalog # GFP-1020 at 1:1,000). Glial cell types (astrocytes/oligodendrocytes/satellite cells) were detected using: rabbit polyclonal anti-ALDH1L1 (Neuromics catalog # RA22119 at 1:200); rabbit polyclonal anti-GFAP (Abcam catalog # ab16997 at 1:100); mouse monoclonal anti-S100 β-subunit (Sigma catalog # S2532 at 1:1,000); mouse monoclonal anti-MBP (BioLegend catalog # 808403 at 1:500). Neuronal cell types were detected using: rabbit monoclonal anti-NeuN (Abcam catalog # ab177487 at 1:300); mouse monoclonal anti-calbindin-D28K (Sigma catalog # C9848 at 1:500); mouse monoclonal anti-neurofilament (SMI-32) (BioLegend catalog # 801701 at 1:500) or by doing a fluorescent Nissl post-staining (NeuroTrace^™^ 530/615; ThermoFisher catalog # N21482 at 1:200 for 20 mins). Sections used as negative controls to differentiate non-specific background signal from specific antibody signal were treated with isotype specific antibodies matching the concentration of the primary antibodies: chicken IgY (BioLegend catalog # 402101 or Abcam catalog # ab50579); mouse IgG (Invitrogen catalog # 31903 or Abcam catalog # ab37355); rabbit IgG (Abcam catalog # ab37415). Following washes in TBS, the sections were incubated in species-specific secondary antibodies conjugated to either Alexa 488 (Jackson Immuno catalog # 703-545-155), 555 (Invitrogen catalog # A31570 or A31572), or 647 (Invitrogen catalog # A31571 or A31573) diluted at 1:1,000 in block buffer for 2 hours at room temperature. Sections were mounted onto slides then treated with TrueBlack^®^ (Biotium, catalog # 23007) diluted in 70% ethanol for 30 seconds to reduce autofluorescence. Slides were rinsed with TBS before mounted in ProLong^™^ Gold Antifade Mountant with DAPI (Invitrogen catalog # P36931). Fluorescent images were acquired using a Zeiss LSM 800 with Airyscan and ZEN Blue software (Zeiss).

The percentage of eGFP-positive cells per cell-type (neurons or glia) was determined in one CNS region (LGN) and one PNS region (DRG), for AAVHSC17 (n = 2 animals), AAVHSC15 (n = 2 animals), and AAVHSC7 (n = 1 animal). All sections used for quantification were acquired with fixed imaging parameters. For the eGFP signal, background was measured from IgY isotype control images and subtracted from the images for quantification. Total neuron or glial counts within a set region of interest were performed with the use of ImageJ or the ZEN Image Analysis module [Region of Interest (ROI) were 500 μm x 500 μm for LGN neuron counts; 200 μm x 200 μm for DRG neuron counts; and 100 μm x 100 μm for LGN and DRG glia counts)]. eGFP-positive cells within each population (neurons or glia) were manually counted. Data is represented as an average of 3 ROIs per image for each capsid with the exception of neuron counts for LGN which was from a single ROI.

### Vector genome analysis in nonhuman primate tissues

All DNA was isolated from fixed tissue using the RecoverAll^™^ Total Nucleic Acid Isolation Kit for formalin- or paraformalin-fixed, paraffin-embedded (FFPE) (ThermoFisher) according to instructions. Paraffin-embedded tissues were deparafinized by xylene treatment followed by an ethanol wash prior to DNA extraction. Deparafinized tissues were protease digested in the provided digestion buffer and bound to a rapid glass-fiber filter, followed by an in-filter RNase treatment. DNA is eluted with 60 μl of 95°C molecular grade water. Vector genome copies per cell were determined by qPCR using TaqMan^™^ Universal PCR Master Mix (ThermoFisher) and eGFP and cyno ApoB specific primers and probes.

eGFP Probe: TACCTGAGCACCCAGTCCGCCCT;

eGFP Forward: CTGCTGCCCGACAACCA;

eGFP Reverse: GACCATGTGATCGCGCTTCT.

Cynomolgus macaque ApoB Probe: CGAGAATCACCCTGCCAGACTTCCAT; Cynomolgus macaque ApoB Forward: TGAAGGTGGAGGACATTCCTCTA; Cynomolgus macaque ApoB Reverse: CTGGAATTGCGATTTCTGGTAA.

## Results

### IV delivery of scAAVHSC-CBA-eGFP results in widespread transduction of the CNS in nonhuman primates

**Fig 1:**
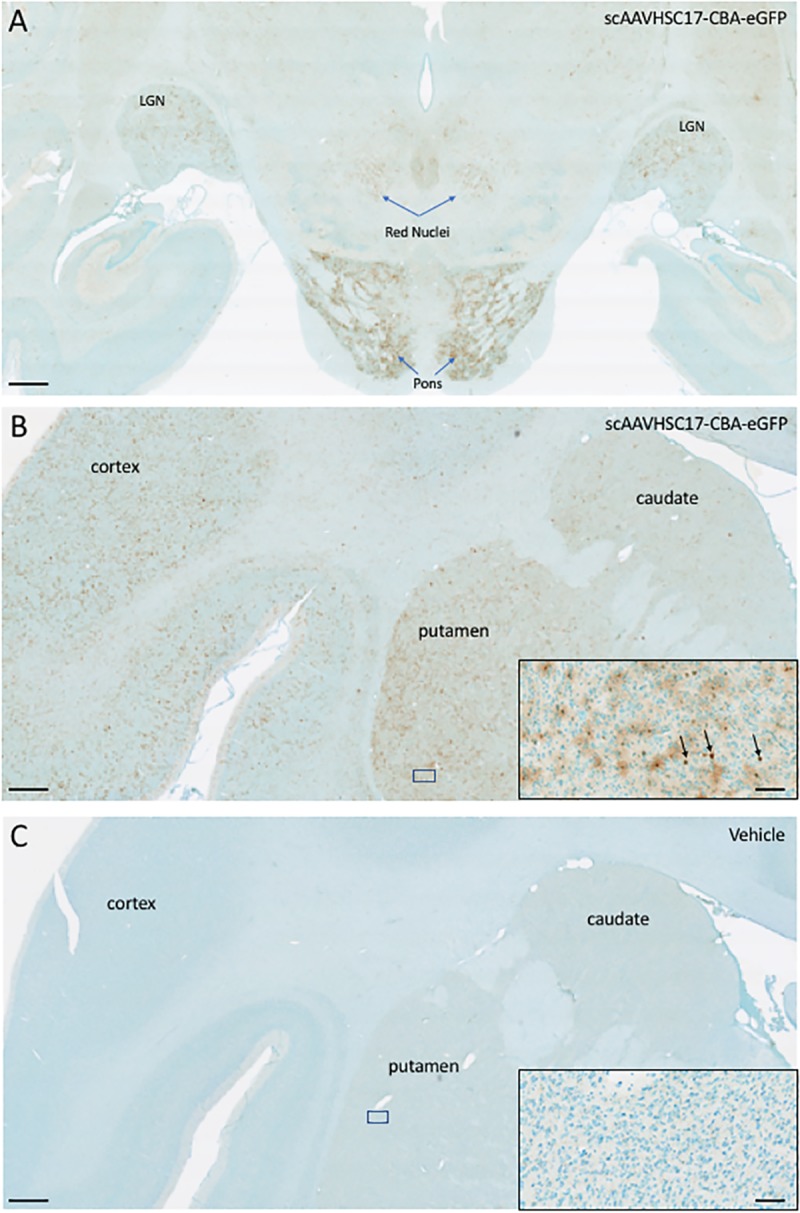
eGFP detection within multiple brain regions of Cynomolgus macaques following IV delivery of scAAVHSC17-CBA-eGFP. (A and B) low power hindbrain and cortical/midbrain regions, respectively, of the two individual macaques (A: animal H16C32; B: animal 16C26) treated with IV scAAVHSC17-CBA-eGFP (1 x10^14^ vg/kg) on Day 0. (C): low power cortical/midbrain view of a macaque treated with IV vehicle alone (animal H16C14). Tissues were harvested two weeks post-dose and brains were sectioned and stained with an anti-eGFP antibody as described under Materials and methods. eGFP was visualized with diaminobenzidine staining (brown color). Location of the cortex, basal ganglia (caudate and putamen) pons, red nuclei and LGN and putamen are shown. Insets are higher magnification views of the boxed areas in each panel. Each bar represents 1000 μm or 50 μm in the insets.

To evaluate biodistribution of AAVHSCs after systemic delivery, self-complementary (sc) AAVHSC7, AAVHSC15 and AAVHSC17 each packaging an eGFP reporter transgene driven by the ubiquitously-expressing chicken beta actin (CBA) promoter were prepared. These capsids were chosen for this work based on biodistribution studies in mice [24]. All animals were negative for serum anti-AAVHSC neutralizing antibodies over a range of sera dilutions. The doses used were the highest possible based on the vector titer, animal body weights at the time of infusion and the volume limits for IV infusion. Each animal received a single IV infusion of either vehicle-alone or vehicle with the indicated scAAVHSC-CBA-eGFP. Two weeks after dosing the animals were humanely euthanized and tissues were harvested for analyses of eGFP expression by IHC and distribution of eGFP vector genomes. Following treatment with scAAVHSC17-CBA-eGFP, eGFP IHC detection was characterized across the whole brain in one monkey to evaluate the coronal biodistribution of eGFP across both hemispheres. eGFP detection was observed throughout the brain in both white and grey matter regions including the cerebral cortex, caudate nuclei and putamen (Fig 1A and 1B). In this animal, detection of eGFP was bilateral in every region examined with high levels of eGFP detected in the pons, red nuclei, and LGN (Fig 1A). Expression in both treated-animals was predominately in glial cells, interpreted to be astrocytic in nature (Fig 1B inset) based on morphology and counterstain. Scattered eGFP-positive single isolated neuronal cell bodies were also seen across these regions (see black arrows in Fig 1B, inset). Astrocytes take on a “fuzzy” brown appearance in these images due to the abundance of highly branched cellular processes and thickness of the tissue sections. No eGFP was detected in animals injected with vehicle alone (Fig 1C). All subsequent IHC was performed on one hemisphere of the brain of each treated animal.

**Fig 2:**
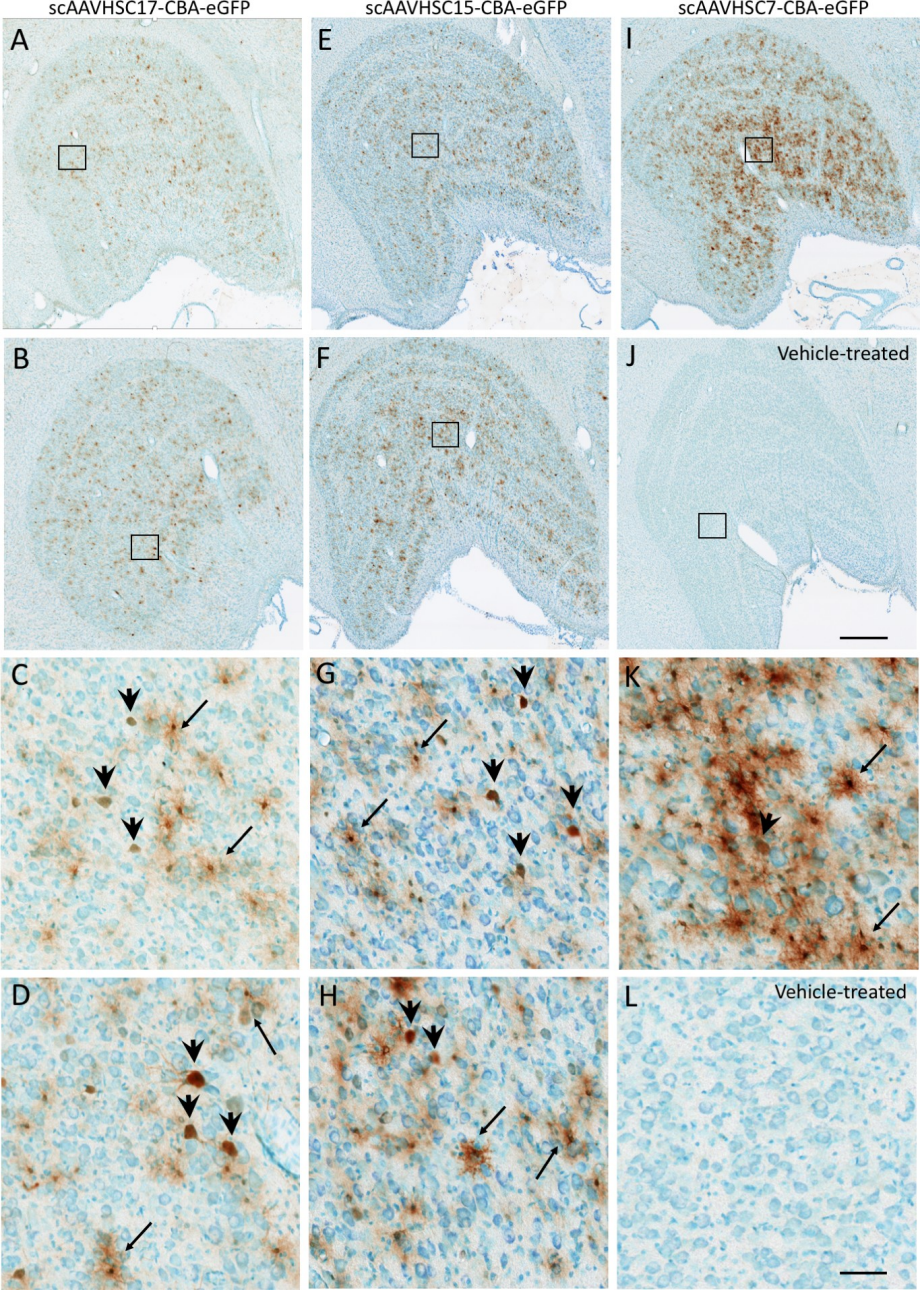
Detection of eGFP expression with neurons and glia of the LGN of Cynomolgus macaques treated with IV scAAVHSC-CBA-eGFP. (A-D) Each animal received an IV injection of scAAVHSC17-CBA-eGFP at 1.0 x10^14^ vg/kg (animals H16C32 and 16C26: A/C and B/D, respectively). (E-H) Each animal received an IV injection of scAAVHSC15-CBA-eGFP at 0.7 x10^14^ vg/kg (animals 16C33 and 16C45: E/G and F/H, respectively). (I and K) The animal received an IV injection of scAAVHSC7-CBA-eGFP [0.7 x10^14^ vg/kg, (animal 16C34)]. (J and L) The animal received an IV injection of an identical volume of vehicle alone (animal H16C14). Tissues were collected and stained for eGFP as described in the legend to Fig 1. Large and small arrows: neuronal and glial staining, respectively. Higher magnification views of the boxed areas in the upper panels are shown in the corresponding lower panels. The bar represents 500 μm (A, B, E, F, I, J) or 50 μm (C, D, G, H, K, L). Brown color represents eGFP staining.

**Fig 3:**
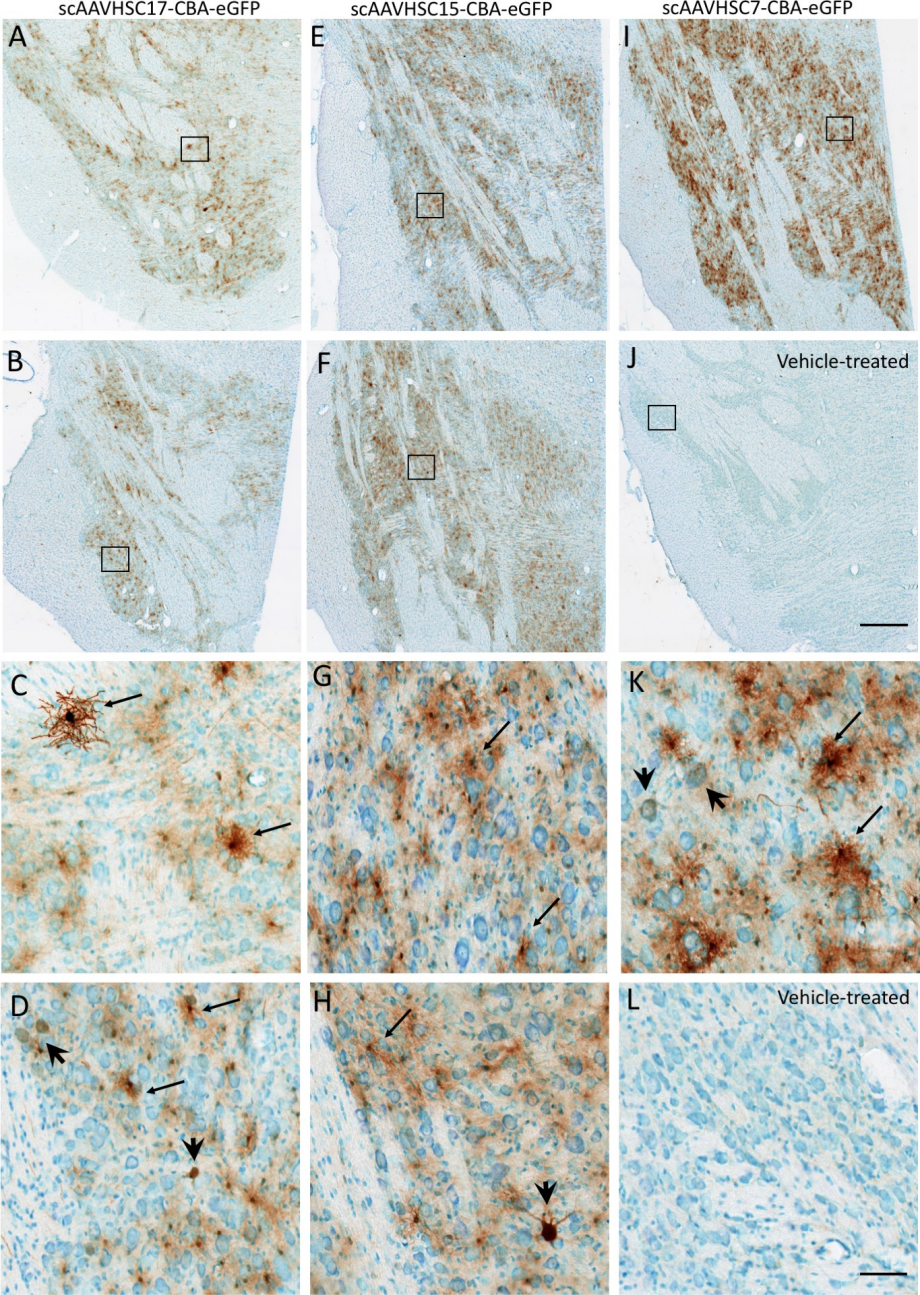
Expression of eGFP within pontine neurons and glia of Cynomolgus macaques treated with an IV injection of scAAVHSC-CBA-eGFP. (A-D) Each animal received an IV injection of scAAVHSC17-CBA-eGFP at 1.0 x 10^14^ vg/kg (animals H16C32 and 16C26: panels A/C and B/D, respectively). (E-H) Each animal received an IV injection of scAAVHSC15-CBA-eGFP at 0.7 x10^14^ vg/kg (animals 16C33 and 16C45: panels E/G and F/H, respectively). (I, K) The animal received an IV injection of scAAVHSC7-CBA-eGFP at 0.7 x10^14^ vg/kg (animal 16C34)]. (J, L) The animal received an IV injection of an equivalent volume of vehicle alone (animal H16C14). Tissues were collected and stained for eGFP as described in the legend to Fig 1. Large and small arrows: neuronal and glial staining, respectively. Higher magnification views of the boxed areas in the upper panels are shown in the corresponding lower panels. The scale bars represent 500 μm (A, B, E, F, I, J) or 50 μm (C, D, G, H, K, L). Brown color represents eGFP staining.

The most pronounced eGFP signal was in the region of the LGN (Fig 2) and pons (Fig 3) for animals treated with systemic scAAVHSC17-CBA-eGFP, scAAVHSC15-CBA-eGFP or scAAVHSC7-CBA-eGFP. The majority of eGFP-positive cells in these brain regions were glial (astrocytes) with occasional neuronal profiles. scAAVHSC7-CBA-eGFP appeared to show greater transduction of astrocytes and neurons within the pons and lateral geniculate nucleus in this animal compared with either scAAVHSC17-CBA-eGFP or scAAVHSC15-CBA-eGFP treated animals (Figs 2 and 3). eGFP-positive cells with astrocyte morphologies showed extensive staining within cell bodies and radial processes in the scAAVHSC7-CBA-eGFP-treated animal (Figs 2K and 3K, S1 Fig). Similarly, the cell bodies, axons and proximal dendritic trees of neurons showed intense eGFP staining in animals treated with scAAVHSC17-CBA-eGFP or scAAVHSC15-CBA-eGFP (Figs 2 and 3, S1 Fig). Within the Purkinje cell layer of the cerebellum, occasional intense eGFP stained Purkinje neuronal cell bodies, axons and corresponding dendritic arbor were observed (S1 Fig). A much larger number of eGFP-positive Bergmann glia cell bodies and radial branches extending through the molecular layer of the cerebellum were observed in animals treated with all three scAAVHSC-CBA-eGFP (S1 Fig for scAAVHSC15-CBA-eGFP). Of note, little or no eGFP staining was seen in endothelial cells of the brain vasculature in any brain region examined in any of the treated-animals (S2 Fig, images from a scAAVHSC17-CBA-eGFP-treated animal are shown). No eGFP positive cells were seen in any of these brain regions in animals treated with vehicle alone (Fig 1C; Fig 2J, Fig 2L, Fig 3J, and Fig 3L).

**Fig 4:**
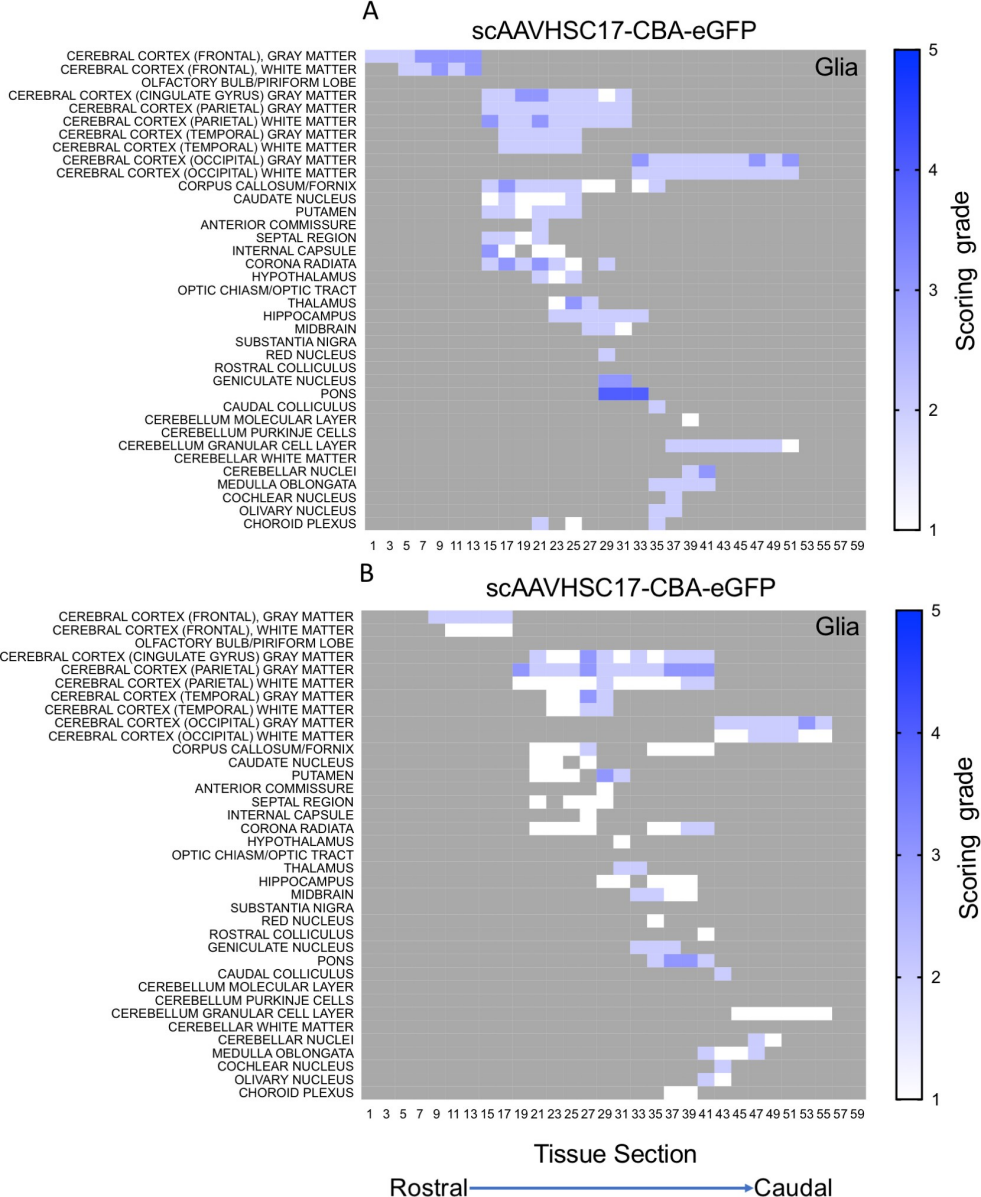
Heat maps of eGFP staining intensity within glia throughout the brain of Cynomolgus macaques treated with IV scAAVHSC17-CBA-eGFP. (A) Glial staining in animal H16C32. (B) glial staining in animal 16C26. All sections were scored in a blinded manner as described under Materials and methods. The percent of cell type stained per structure on each slide was grade 1: <1%, grade 2: 1–5%, grade 3: 5–15%, grade 4: 15–40%, and grade 5: >40%. Grey areas represent those areas where either no brain structure was present or no eGFP staining was seen. The numbers below each heat map represent the coronal brain section from most rostral (slide 1) to most caudal (slide 59).

**Fig 5:**
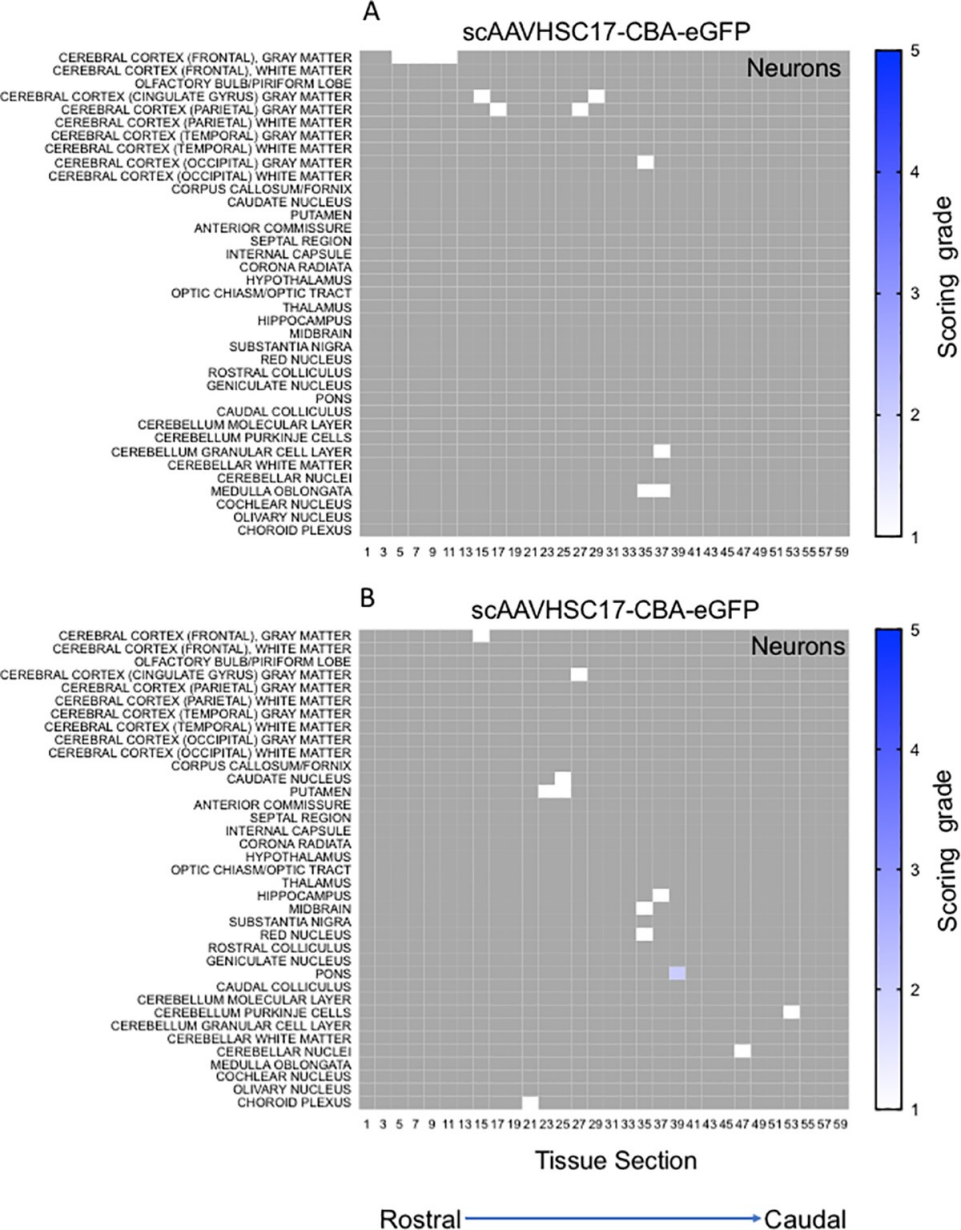
Heat maps of eGFP staining intensity within neurons throughout the brain of Cynomolgus macaques treated with IV scAAVHSC17-CBA-eGFP. (A) Neuronal staining in animal H16C32. (B) Neuronal staining in animal 16C26. All sections were scored in a blinded manner as described under Materials and methods. The percent of cell type stained per structure on each slide was grade 1: <1%, grade 2: 1–5%, grade 3: 5–15%, grade 4: 15–40%, and grade 5: >40%. Grey areas represent those areas where either no brain structure was present or no eGFP staining was seen. The numbers below each heat map represent the coronal brain section from most rostral (slide 1) to most caudal (slide 59).

**Fig 6:**
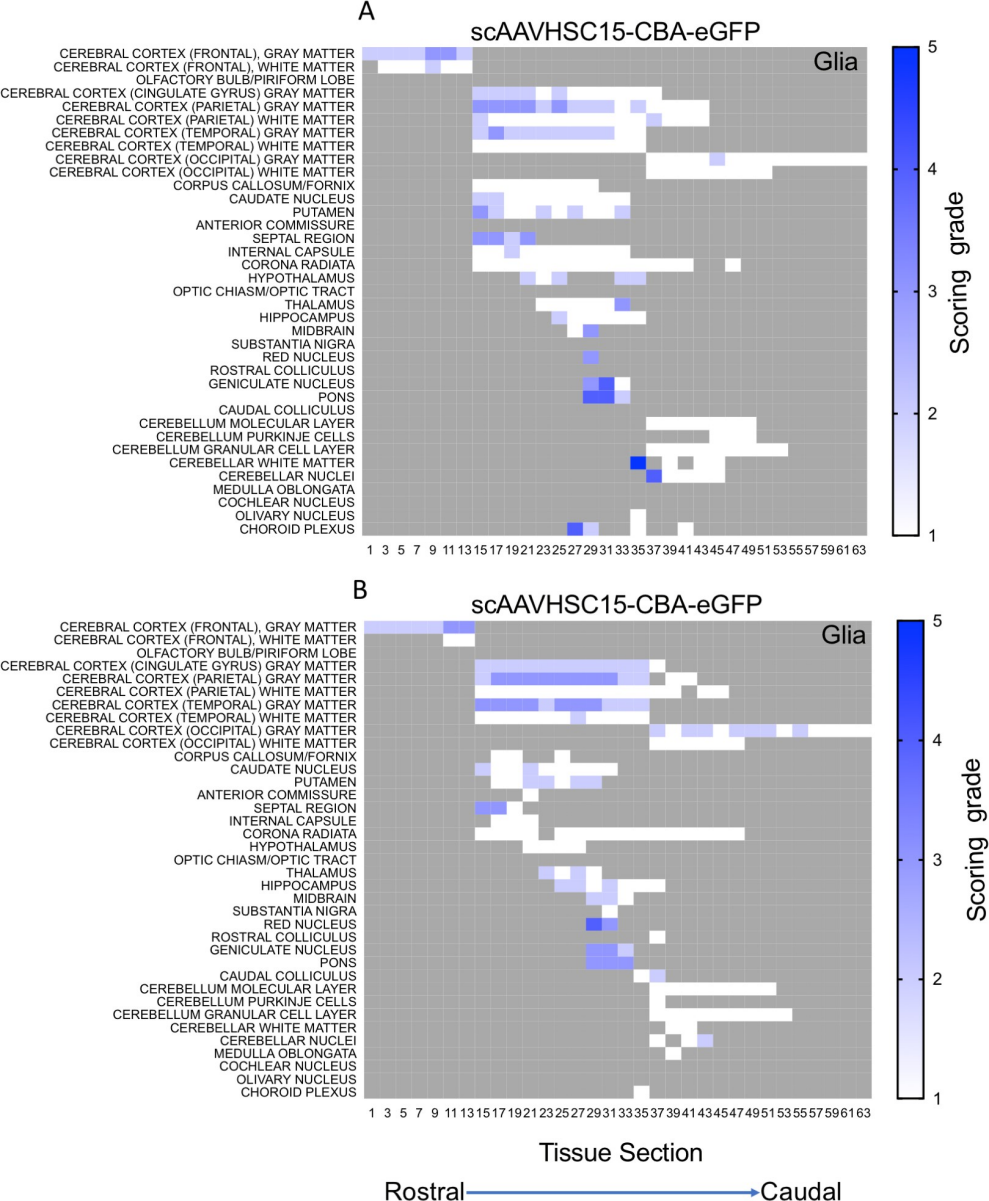
Heat maps of eGFP staining intensity within glia throughout the brain of Cynomolgus macaques treated with IV scAAVHSC15-CBA-eGFP. (A) Glial eGFP staining in animal 16C33. (B) Glial staining in animal 16C45. All sections were scored in a blinded manner as described under Materials and methods. The percent of cell type stained per structure on each slide was grade 1: <1%, grade 2: 1–5%, grade 3: 5–15%, grade 4: 15–40%, and grade 5: >40%. Grey areas represent those areas where either no brain structure was present or no eGFP staining was seen. The numbers below each heat map represent the brain coronal section from most rostral (slide 1) to most caudal (slide 63).

**Fig 7:**
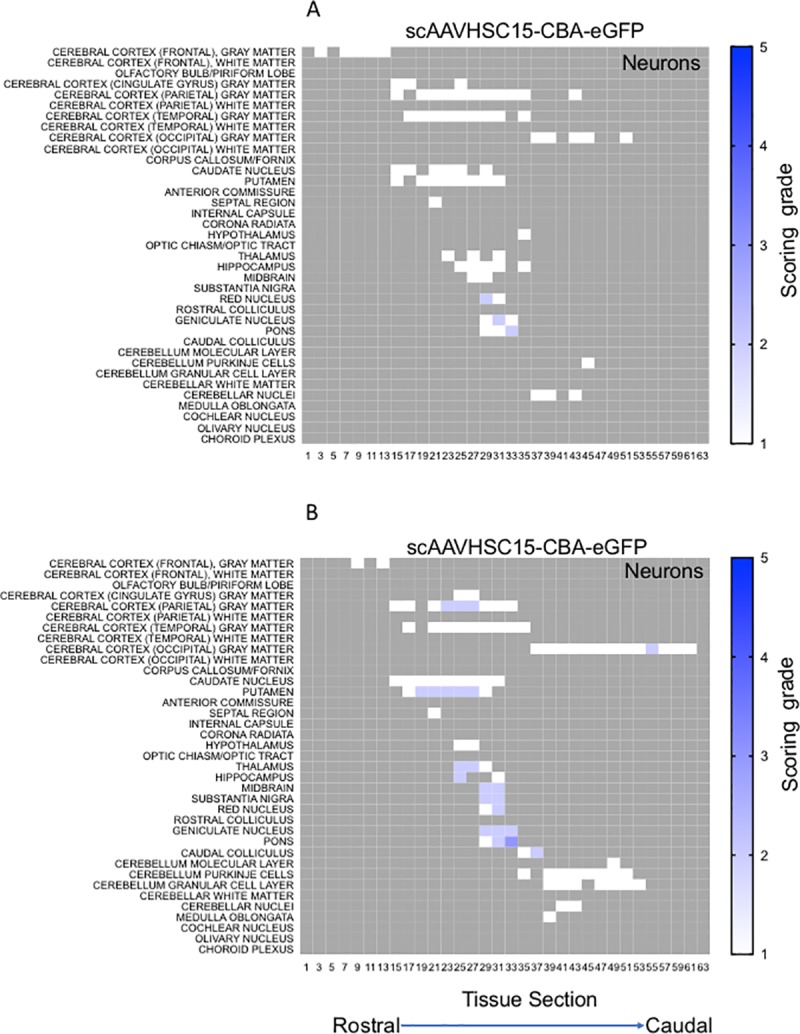
Heat maps of eGFP staining intensity within neurons throughout the brain of Cynomolgus macaques treated with IV scAAVHSC15-CBA-eGFP. (A) Neuronal eGFP staining in animal 16C33. (B) Neuronal staining in animal 16C45. All sections were scored in a blinded manner as described under Materials and methods. The percent of cell type stained per structure on each slide was grade 1: <1%, grade 2: 1–5%, grade 3: 5–15%, grade 4: 15–40%, and grade 5: >40%. Grey areas represent those areas where either no brain structure was present or no eGFP staining was seen. The numbers below each heat map represent the brain coronal section from most rostral (slide 1) to most caudal (slide 63).

The distribution and extent of eGFP staining across the brain presented in a rostro-caudal manner. Corresponding eGFP-positive regions were scored in a blinded fashion. The evaluation was performed to determine 1) the most prominent eGFP-positive cerebral regions and 2) the corresponding percent of eGFP-positive cell type (neuron or glia)/structure in said regions. The grading scheme was designed to allow for the differentiation of subtle staining (<1% of the cell type/structure in a given defined anatomic region stained for eGFP) from areas with more pronounced staining. At the higher grades [4 (15–40% of the cell type/structure and 5 (>40% of the cell type/structure)] there is the possibility of overlap because the grades were estimated based on qualitative visual observation. Heat maps of these data show eGFP staining in glial profiles in virtually all brain regions scored for both animals treated with scAAVHSC17-CBA-eGFP (Fig 4A and 4B) with minimal amounts of eGFP-positive neurons throughout the brain (Fig 5A and 5B). Glial eGFP staining was highest in the frontal, parietal and temporal cortex, red nucleus, pons, LGN and the septal region with a similar distribution and intensity of staining observed in the two animals treated with scAAVHSC17-CBA-eGFP (Fig 4A and 4B). The distribution of eGFP-positive neurons was generally similar but much less pronounced to that of glial cell staining. A similar pattern of eGFP staining within glial cells was observed in the brain of animals treated with a single IV dose of scAAVHSC15-CBA-eGFP (Fig 6A and 6B). Treatment with scAAVHSC15-CBA-eGFP led to a somewhat higher level and distribution of eGFP-positive neuronal profiles in the brain regions analyzed herein compared to treatment with scAAVHSC17-CBA-eGFP (Fig 7A and 7B vs. Fig 5A and 5B).

**Fig 8:**
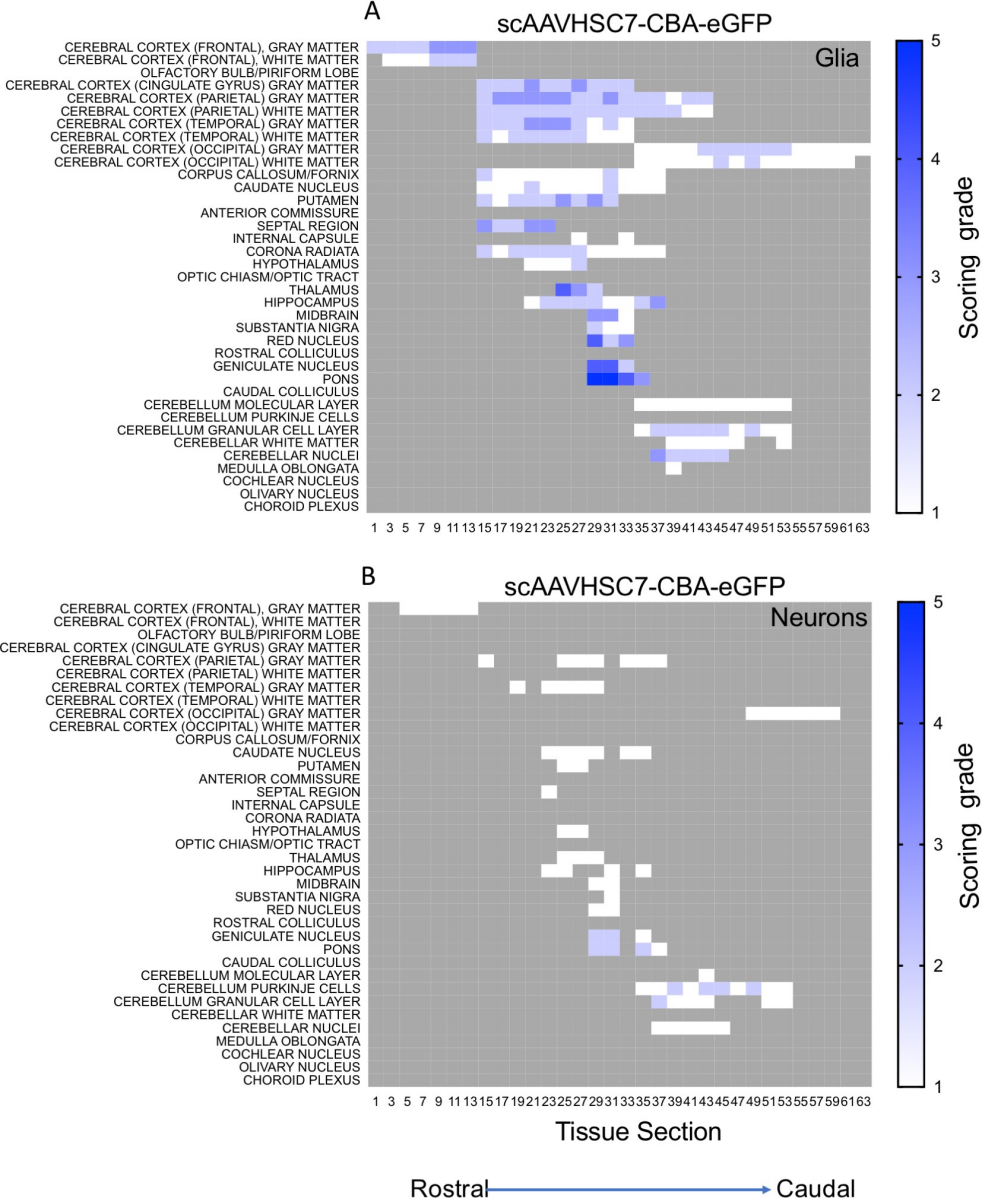
Heat maps of eGFP staining intensity within glia and neurons throughout the brain of Cynomolgus macaques treated with IV scAAVHSC7-CBA-eGFP. (A) Glial staining in animal 16C34. (B) Neuronal staining in animal 16C34. All sections were scored in a blinded manner as described under Materials and methods. The percent of cell type stained per structure on each slide was grade 1: <1%, grade 2: 1–5%, grade 3: 5–15%, grade 4: 15–40%, and grade 5: >40%. Grey areas represent those areas where either no brain structure was present or no eGFP staining was seen. The numbers below each heat map represent the brain coronal section from most rostral (slide 1) to most caudal (slide 63).

Similarly, an IV dose of scAAVHSC7-CBA-eGFP resulted in a broad, intense distribution of astrocytic-like eGFP staining and a neuronal distribution of GFP staining similar to that observed following dosing with scAAVHSC15-CBA-eGFP Overall, eGFP-positive cells were mainly, but not exclusively, glial in nature in these scAAVHSC15-CBA-eGFP- or scAAVHSC7-CBA-eGFP-treated animals (Fig 6A and 6B, Fig 7A and 7B, and Fig 8A and 8B).

**Fig 9:**
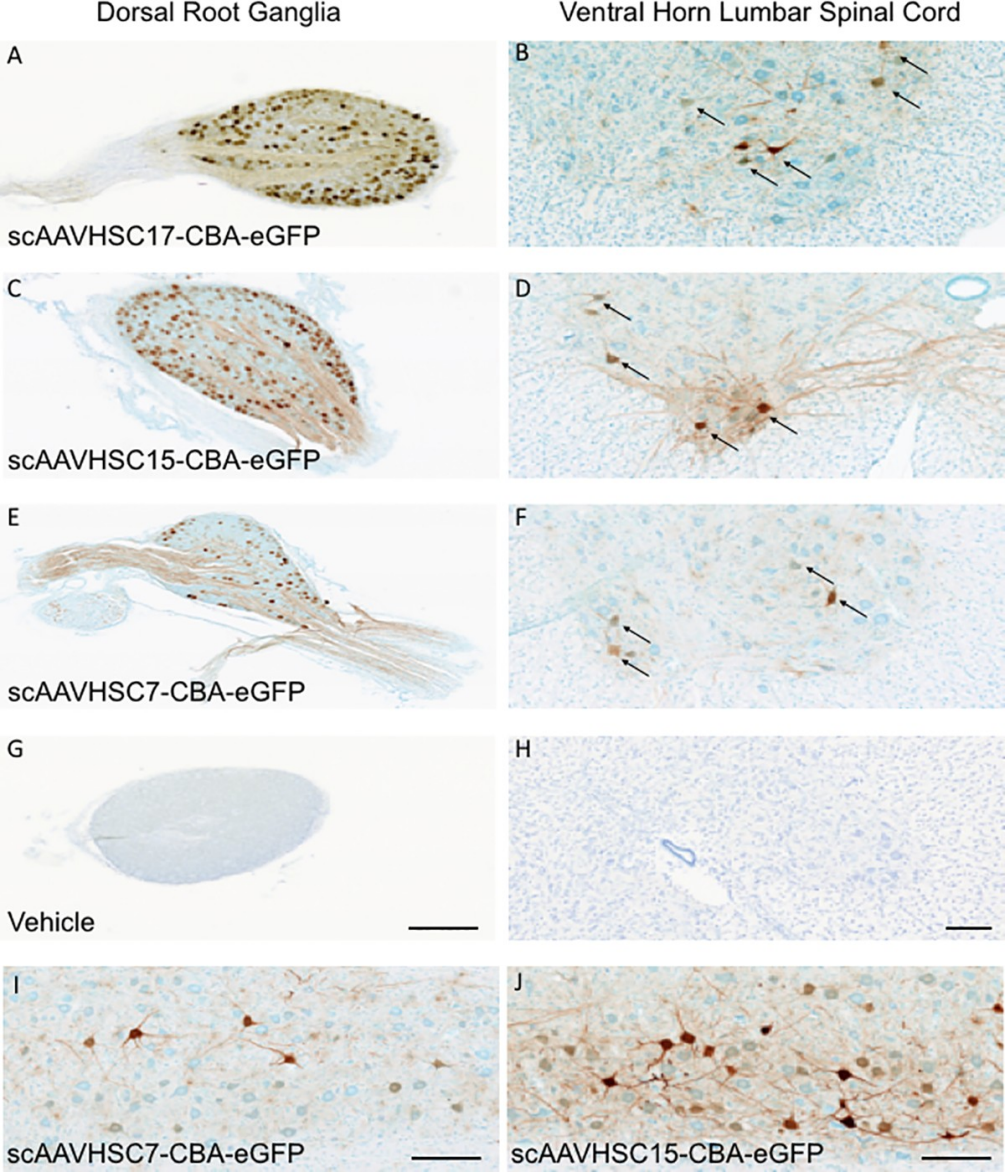
eGFP staining within the DRG and spinal cord in Cynomolgus macaques treated with IV scAAVHSC-CBA-eGFP. (A,B) Animal treated with an IV dose of scAAVHSC17-CBA-eGFP at 1.0 x 10^14^ vg/kg. (C,D) Animal treated with an IV dose of scAAVHSC15-CBA-eGFP at 0.7 x 10^14^ vg/kg. (E,F) Animal treated with scAAVHSC7-CBA-eGFP at 0.7 x 10^14^ vg/kg. (G,H) Animal treated with an IV injection of vehicle alone. eGFP staining in lumbar DRG and cross-sections of lumbar spinal cords are shown in the left and right panels, respectively. The bar represents 500 μm (A, C, E and G) and 50 μm (B, D, F and H). (I) Longitudinal section of lumbar spinal cord from an animal treated with scAAVHSC7-CBA-eGFP. (J) Longitudinal section of lumbar spinal cord from an animal treated with scAAVHSC15-CBA-eGFP. The scale bars in I and J represent 250 μm. Arrows = motor neurons.

**Fig 10:**
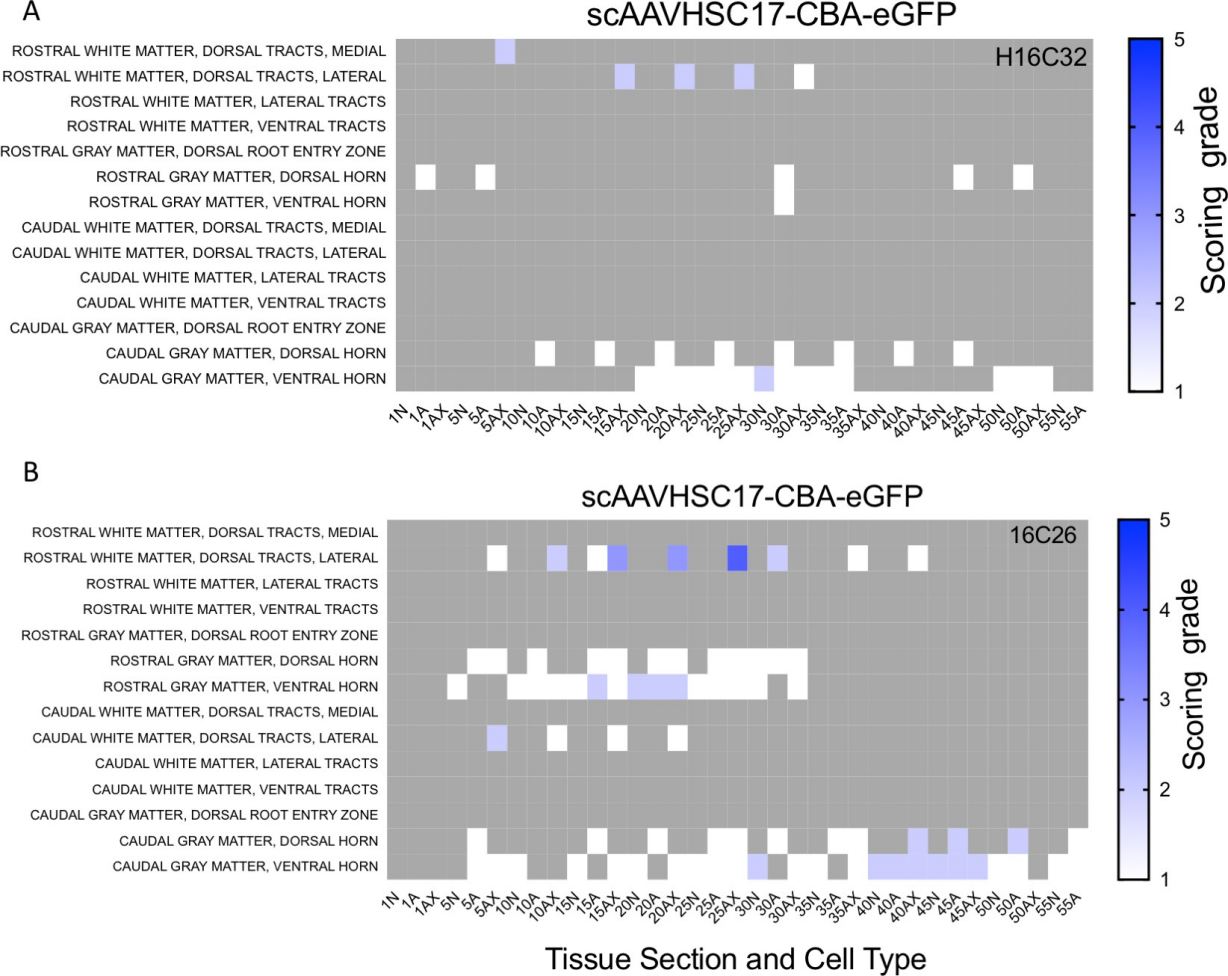
Heat maps of eGFP staining intensity within glia, neurons and neuronal axons in spinal cords of Cynomolgus macaques treated with IV scAAVHSC17-CBA-eGFP. (A) Animal H16C32 received an IV injection of scAAVHSC17-CBA-eGFP. (B) Animal 16C26 was treated with an IV injection of scAAVHSC17-CBA-eGFP. Each animal was dosed at 1.0 x 10^14^ vg/kg. All sections were scored in a blinded manner as described under Materials and methods. Only the rostral and caudal portions of the spinal cords were available for analysis. The percent of cell type stained per structure on each slide was grade 1: <1%, grade 2: 1–5%, grade 3: 5–15%, grade 4: 15–40%, and grade 5: >40%. Grey areas represent those areas where either no cord structure was present or no eGFP staining was seen. The numbers below each heat map represent the spinal cord sections with N = neurons, Ax = axons and A = glia. Animal identifiers are in the upper right of each panel.

**Fig 11:**
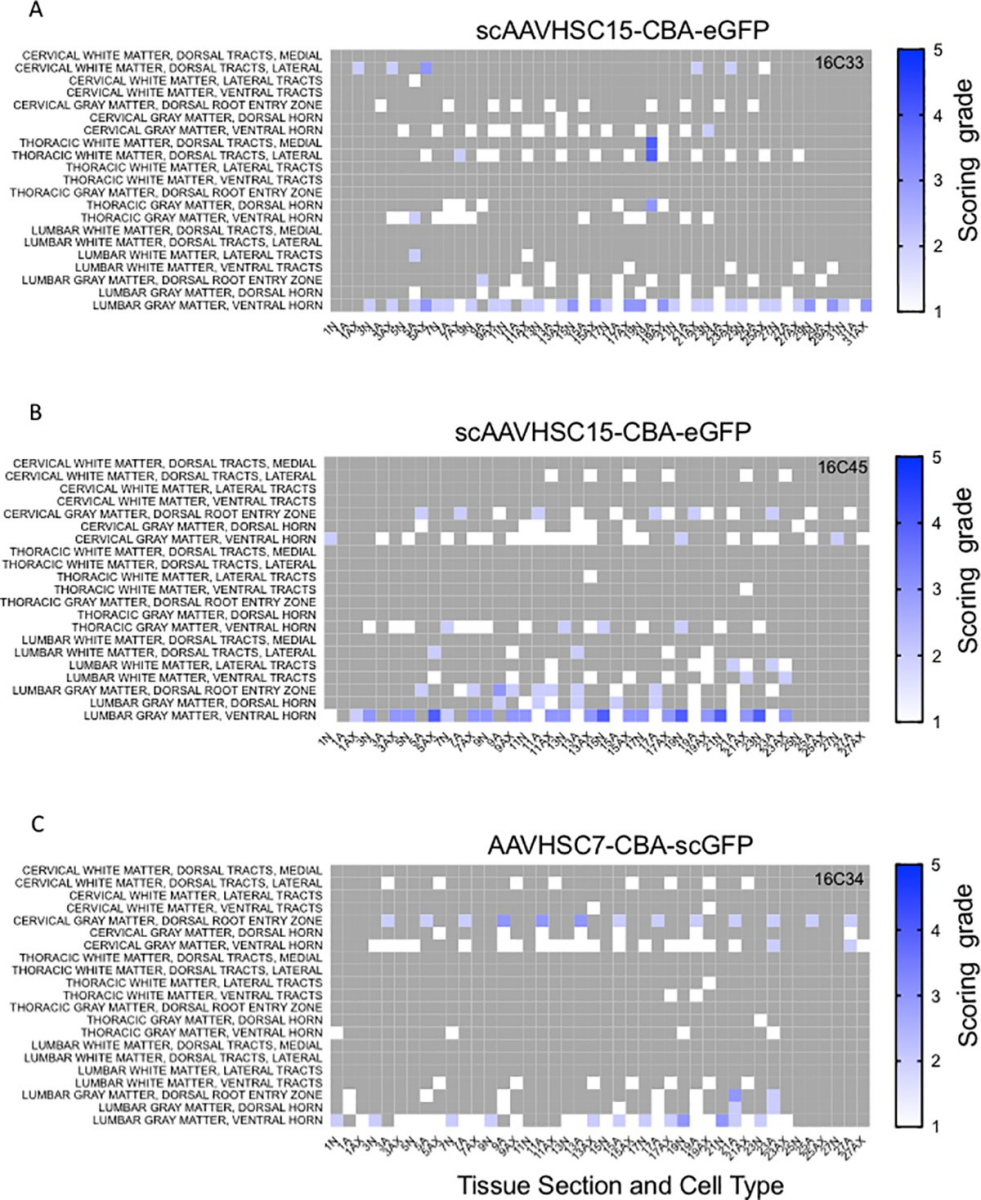
Heat maps of eGFP staining intensity within glia, neurons and neuronal axons in spinal cords of Cynomolgus macaques treated with IV scAAVHSC-CBA-eGFP. (A) Animal 16C33 was treated with an IV injection of scAAVHSC15-CBA-eGFP. (B) Animal 16C45 received an IV injection of scAAVHSC15-CBA-eGFP. (C) Animal 16C34 was treated with an IV injection of scAAVHSC7-CBA-eGFP. All animals were dosed at 0.7 x 10^14^ vg/kg. All sections were scored in a blinded manner as described under Materials and methods. The percent of cell type stained per structure on each slide was grade 1: <1%, grade 2: 1–5%, grade 3: 5–15%, grade 4: 15–40%, and grade 5: >40%. Grey areas represent those areas where either no cord structure was present or no eGFP staining was seen. The numbers below each heat map represent the spinal cord sections with N = neurons, Ax = axons and A = glia. Animal identifiers are in the upper right of each panel.

In addition to widespread transduction of the brain, eGFP staining was detected in neurons in the sensory ganglia (Fig 9A, 9C and 9E) and spinal cord (Fig 9B, 9D and 9F) following a systemic dose of scAAVHSC17-CBA-eGFP (Fig 9A and 9B), scAAVHSC15-CBA-eGFP (Fig 9C and 9D) or scAAVHSC7-CBA-eGFP (Fig 9E and 9F). Longitudinal sections of spinal cords were also taken from animals treated with either scAAVHSC7-CBA-eGFP (Fig 9I) or scAAVHSC15-CBA-eGFP (Fig 9J) which showed eGFP-positive cells with large motor neurons of the ventral horn. Heat maps of the distribution and staining intensity within the spinal cords are shown in Fig 10A and 10B and Fig 11A–11C. For all treated animals, eGFP-positive cells were localized within large motor neurons (cell bodies, dendritic arbors and axons) and in glia-like profiles (seldom in interneurons) in multiple regions of the spinal cord. There was occasional pronounced eGFP staining in glial cells of the dorsal medial and lateral tracts. It should be noted that for animals treated with scAAVHSC17-CBA-eGFP only spinal cord samples from the upper third (rostral in the figure) and lower third (caudal in the figure) were collected and processed. No eGFP-positive cells were seen in the spinal cord of animals treated with vehicle alone (Fig 9H).

eGFP staining was extensive in the sensory neurons of the sensory ganglia (DRG) of all diameter, in some of the satellite (glial) cells abutting the immuno-positive neuronal cell bodies and present in proximal axons of the nerve and dorsal root in animals treated with scAAVHSC17-CBA-eGFP, scAAVHSC15-CBA-eGFP or scAAVHSC7-CBA-eGFP (Fig 9A, 9C and 9E, respectively). Of the sensory neuronal axons labelled intensively, we could not differentiate between axonal profiles of the sensory neurons or whether the oligodendrocytes that myelinate some of these sensory (Aβ) axons were eGFP positive. The DRGs of animals treated with vehicle alone were negative for eGFP staining (Fig 9G).

**Fig 12:**
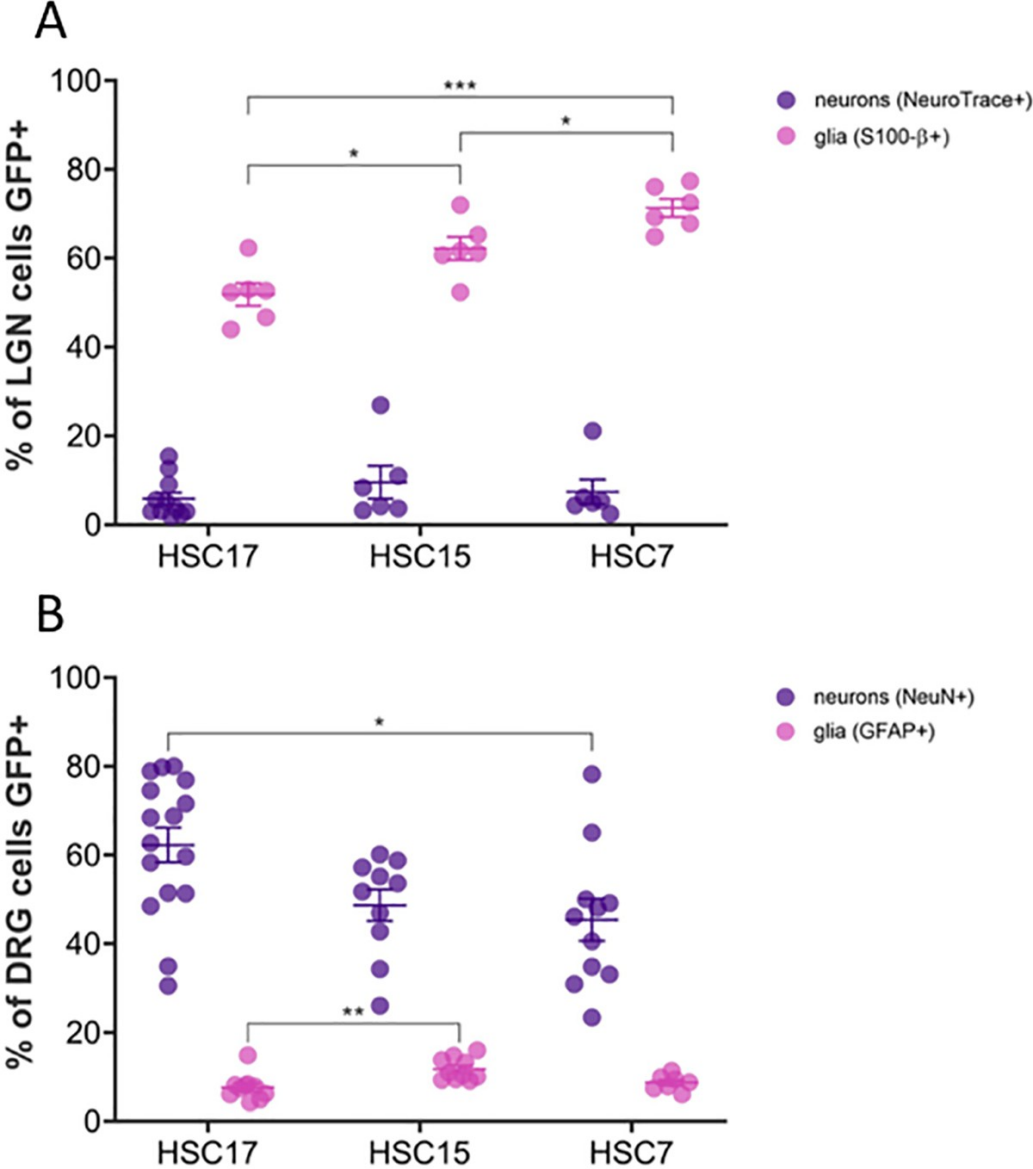
Variation in the transduction efficiency of glia and neurons in the central and peripheral nervous systems. (A) Quantitation of eGFP immunostaining in the LGN of the CNS [S100-β+ (glia); *n* = 6 sections of tissue from 1–2 animals/capsid; NeuroTrace+ (neurons); *n* = 6–11 sections of tissue from 1–2 animals/capsid]. (B) Quantitation of eGFP staining in the DRG of the PNS [GFAP+ (glia); *n* = 7–10 sections of tissue from 1–2 animals/capsid; (NeuN+ (neurons); *n* = 10–16 sections of tissue from 1–2 animals/capsid). For scAAVHSC7-CBA-eGFP, tissue from one animal was stained. Individual data points are shown along with mean ± SEM. *p < 0.05; **p < 0.005; ***p < 0.001; one-way ANOVA with Tukey’s multiple comparisons test. All glia counts were within 100 μm x 100 μm areas and DRG neuron counts were within 200 μm x 200 μm. For these, each data point represents the average of counts from 3 ROIs within a single image. For neuron counts in LGN, each data point represents counts from an entire single image (500 μm x 500 μm). HSC17 = scAAVHSC17-CBA-eGFP, HSC15 = scAAVHSC15-CBA-eGFP, HSC7 = scAAVHSC7-CBA-eGFP.

The distribution of eGFP staining between glia and neurons in the central (LGN) and peripheral (DRG) nervous systems were assessed in co-labeling studies using glial (S100-β and GFAP) and neuronal (NeuroTrace and NeuN) markers (Fig 12). Both these regions were chosen as they emerged as common regions of high eGFP staining between the capsids. In the LGN, 52%, 62% and 71% of S100-β-labeled glial cells were eGFP- positive, on average, in animals dosed with scAAVHSC17-CBA-eGFP, scAAVHSC15-CBA-eGFP and scAAVHSC7-CBA-eGFP, respectively (Fig 12A, pink symbols). For all vectors tested, approximately 6–10% of NeuroTrace-positive neurons were also eGFP-positive (Fig 12A, purple symbols). Overall the eGFP staining scores were below those quantified by immunofluorescense combined with confocal microscopy. We attribute these differences to sensitivity of the assays utilized to provide the associated scores. Within the DRG of the PNS, approximately 62%, 49%, and 45% of NeuN-positive neurons were eGFP-positive in animals dosed with scAAVHSC17-CBA-eGFP, scAAVHSC15-CBA-eGFP and scAAVHSC7-CBA-scGFP (Fig 12B, purple symbols) and about 8–9% of GFAP-positive glial cells were eGFP-positive (Fig 12B, pink symbols).

**Fig 13:**
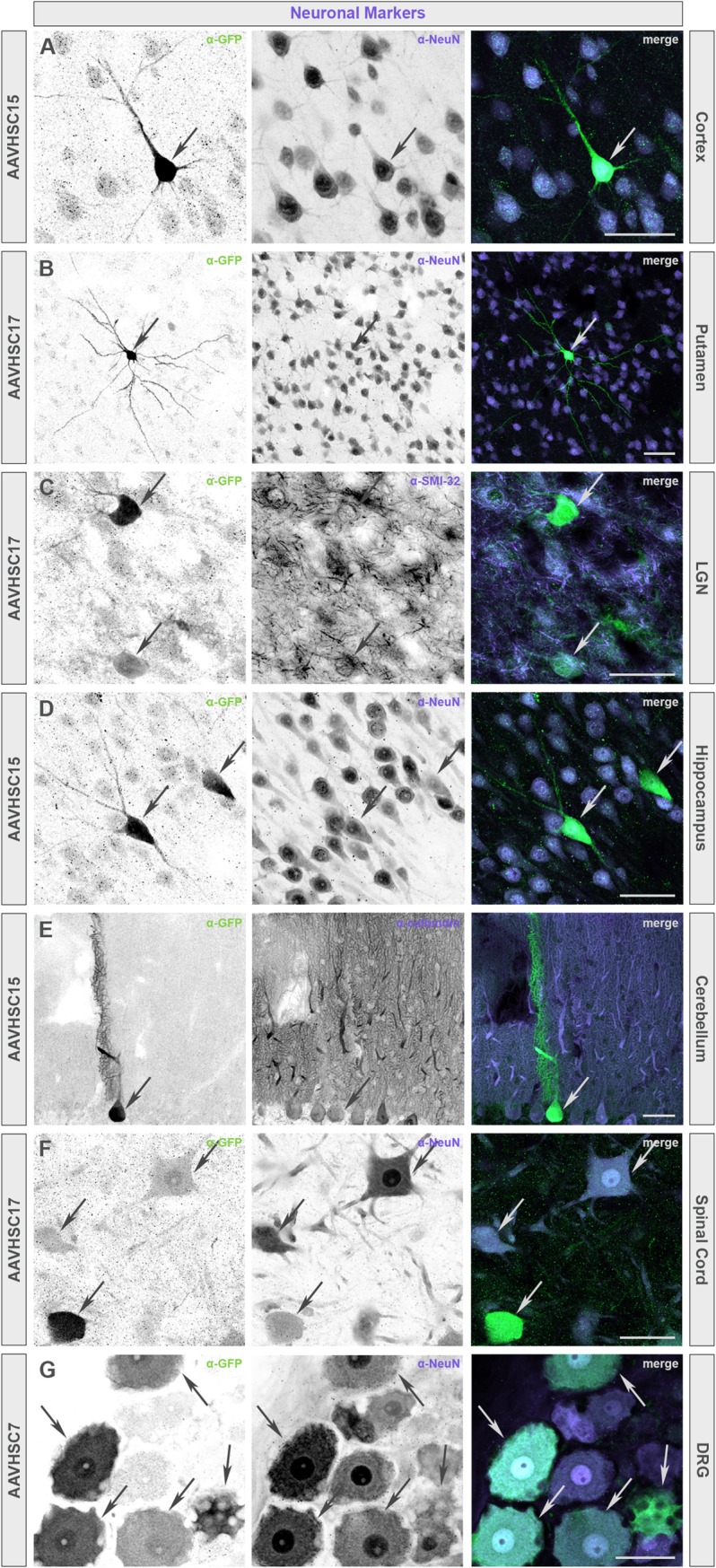
Colocalization of eGFP immunofluorescence with neuronal markers in multiple brain regions of nonhuman primates treated with an IV dose of scAAVHSC-CBA-eGFP. (A, D, E) Animals received an intravenous dose of scAAVHSC15-CBA-eGFP. (B, C, F) Animals received an intravenous dose of scAAVHSC17-CBA-eGFP. (G) The animal received an intravenous dose of scAAVHSC7-CBA-eGFP. eGFP-positive cells exhibiting neuronal-like profiles co-labeled with the neuronal markers NeuN (A, B, D, F, G), neurofilament H (SMI-32) (C), or calbindin (E). In all panels, arrows indicate colocalization. Scale bars represent 50 μm.

**Fig 14:**
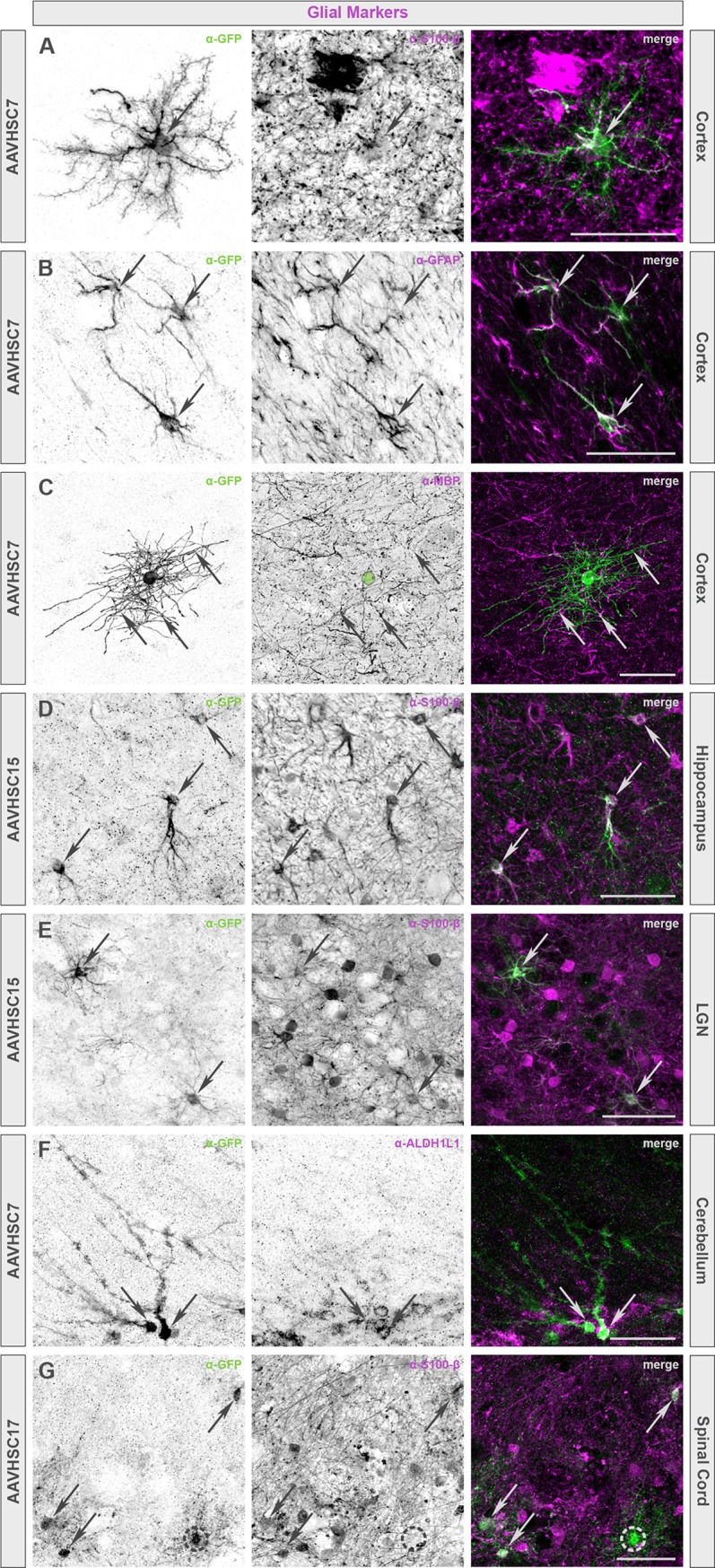
Colocalization of eGFP immunofluorescence with glial markers in multiple brain regions of nonhuman primates treated with an IV dose of scAAVHSC-CBA-eGFP. (A, B, C, F) Animals were dosed with scAAVHSC7-CBA-eGFP. (D, E) Animals were dosed with scAAVHSC15-CBA-eGFP. (G) The animal was dosed with scAAVHSC17-CBA-eGFP. Glial-like profiles of eGFP-positive cells included protoplasmic and fibrous astrocytes (A, B and D-G, respectively) as well as oligodendrocytes (C). The identity of cells with these profiles was confirmed with either myelin basic protein (MBP), a marker exclusively expressed by myelinating glia including oligodendrocytes (C) or astrocyte markers, including S100-β (A, D, E, G), glial fibrillary acidic protein (GFAP) (B), and ALDH1L1 (F). In all panels, arrows indicate colocalization. Green circle in C denotes the location of the oligodendrocyte cell body. Dashed circle in G marks an eGFP-positive cell with a glial-like profile that has no S100-β expression. Scale bars represent 50 μm.

To identify neuronal and glial cell types transduced by the AAVHSCs in nonhuman primates, sections of the brain and spinal cord were prepared and sequentially stained for eGFP and markers for neurons (NeuN, SMI-32, or calbindin), oligodendrocytes (MBP), or astrocytes (GFAP, S100-β or ALDH1L1). Representative sections of animals treated with scAAVHSC17-CBA-eGFP, scAAVHSC15-CBA-eGFP, or scAAVHSC7-CBA-eGFP are shown (Figs 13 and 14). In these animals, cell bodies and dendrites that stained positive for eGFP co-stained with markers for neurons within each of the brain regions examined including the cortex, putamen, LGN, hippocampus, and cerebellum (Fig 13A–13E). In the spinal cord, large motor neurons within the ventral horn were co-labeled with antibodies to eGFP and NeuN (Fig 13F) and in the DRG, cell bodies of sensory neurons were strongly positive for both eGFP and NeuN (Fig 13G) alongside a few eGFP-positive satellite cells. Similar staining profiles were observed for each of the scAAVHSC-CBA-eGFP tested. Within the cortex, highly branched, eGFP-positive cells were co-labeled with the astrocyte markers S100-β and GFAP (Fig 14A and 14B, respectively) as well as the oligodendrocyte marker MBP (Fig 14C). Similarly, in the hippocampus, LGN and spinal cord, eGFP-positive cells co-stained with S100-β confirming their astrocyte profiles (Fig 14D, 14E and 14G, respectively). Within the cerebellum, the cell bodies and radial processes characteristic of Bergmann glial cells were stained for eGFP and the astrocyte marker ALDH1L1 (Fig 14F). These data demonstrate that the AAVHSCs have tropism for astrocytes, oligodendrocytes, satellite cells and neurons throughout the central and peripheral nervous systems following systemic delivery in nonhuman primates.

**Fig 15:**
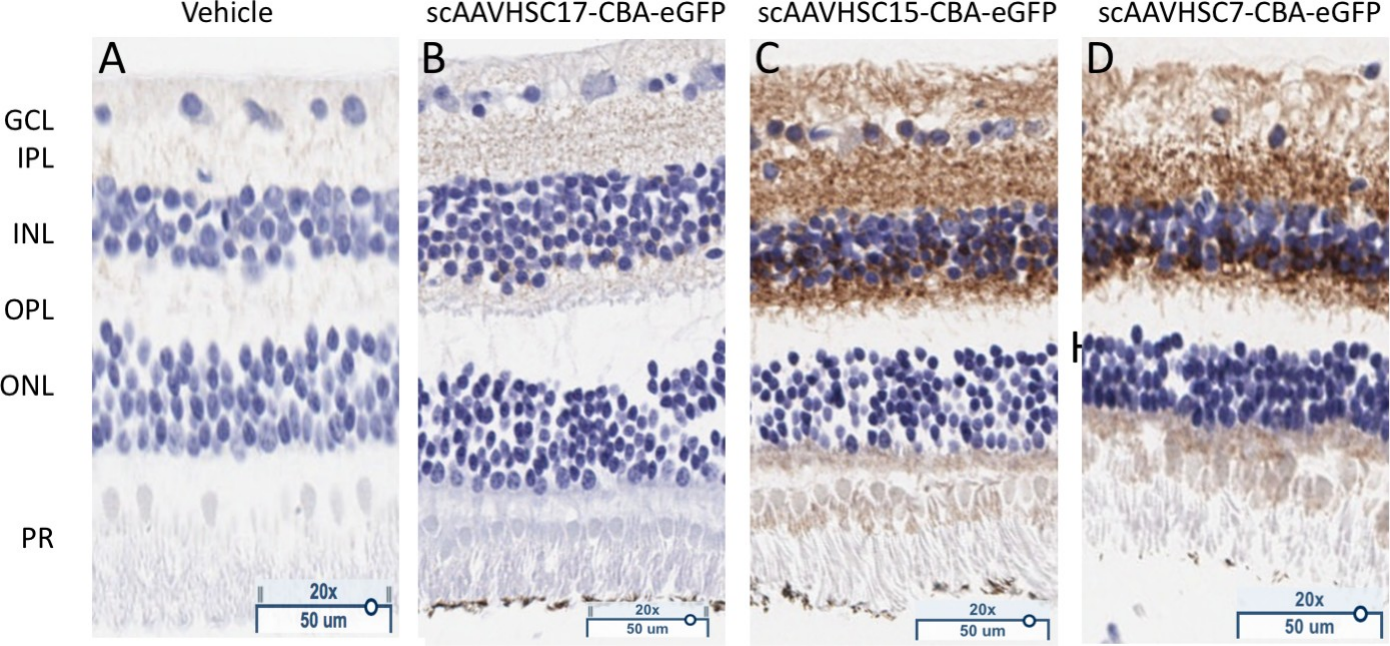
eGFP staining in the retinas of Cynomolgus macaques following IV delivery of scAAVHSC-CBA-eGFP. (A) Animals received an IV dose of vehicle alone. (B) The animal was treated with an IV dose of scAAVHSC17-CBA-eGFP. (C) The animal was treated with an IV dose of scAAVHSC15-CBA-eGFP. (D) The animal received an IV dose of scAAVHSC7-CBA-eGFP. Tissues were harvested two weeks post-dosing and processed for eGFP staining as described under Materials and methods. Brown color represents eGFP detection. Tissue sections were counterstained with thionine. PR = photoreceptor layer, ONL = outer nuclear layer, OPL = outer plexiform layer, INL = inner nuclear layer, IPL = inner plexiform layer, GCL = ganglion cell layer.

Within the retinas of macaques treated with IV scAAVHSC15-CBA-eGFP and scAAVHSC7-CBA-eGFP, the cells in the retinal ganglion cell layer and their afferent fibers, processes within the inner plexiform layer and the inner nuclear layer showed eGFP staining (Fig 15C and 15D). In the inner nuclear layer, it was not evident as to whether neuronal and glial (Müller) profiles were equally eGFP-positive, albeit their endfeet appeared stained in the retinal ganglion cell layer. In contrast, relatively little eGFP staining was apparent in the retina of animals treated with vehicle alone or in the retina of animals treated with IV scAAVHSC17-CBA-eGFP (Fig 15A and 15B, respectively). In all animals, the observed eGFP staining was bilateral.

### IV delivery of scAAVHSC-CBA-eGFP results in transduction of peripheral tissues in nonhuman primates

**Fig 16:**
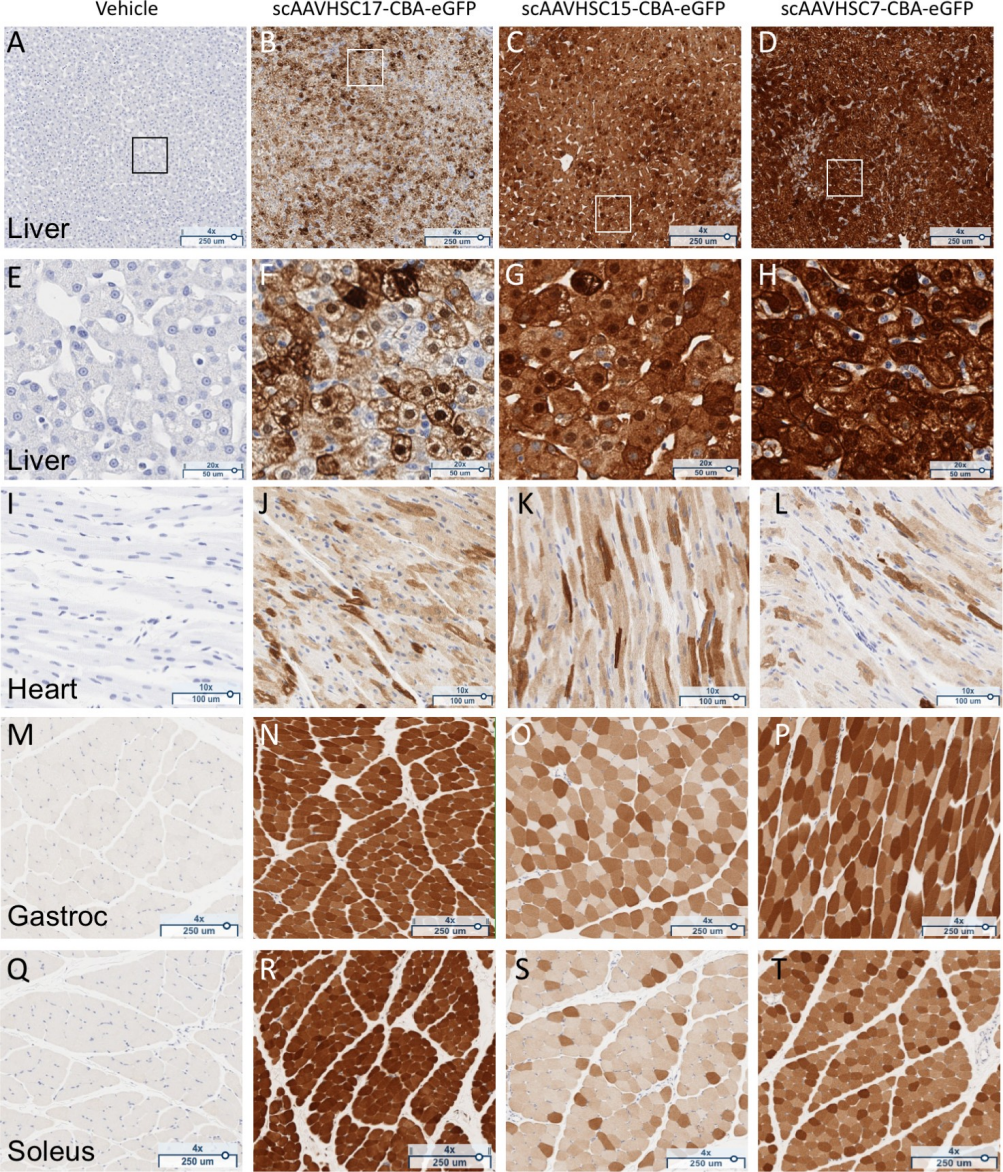
Widespread eGFP staining in peripheral tissues of nonhuman primates dosed with scAAVHSC-CBA-eGFP. (A, E, I, M, Q) The animal received an IV dose of vehicle alone. (B, F, J, N, R) The animal received an IV dose of scAAVHSC17-CBA-eGFP. (C, G, K, O, S) The animal received an IV dose of scAAVHSC15-CBA-eGFP. (D, H, L, P, T) The animal received an IV dose of scAAVHSC7-CBA-eGFP. Tissues were harvested two weeks post-dose and were processed for eGFP staining as described under Materials and methods. Representative tissues shown are: liver (A-D) with higher magnification views of boxed areas shown in E-H; cardiac muscle (I-L), gastrocnemius muscle (M-P), and soleus muscle (Q-T). Brown staining represents eGFP staining.

A single IV injection of scAAVHSC17-CBA-eGFP, scAAVHSC15-CBA-eGFP or scAAVHSC7-CBA-eGFP led to extensive transduction of the liver (Fig 16B–16D and 16F–16H), cardiac muscle (Fig 16J–16L) and skeletal muscle [Fig 16N–16P (gastrocnemius) and Fig 16R–16T (soleus)] of these animals. No eGFP signal was seen in tissues from animals treated with vehicle alone (Fig 16A, 16E, 16I, 16M and 16Q). In liver, there was stronger periportal eGFP signal compared with eGFP expression in the centrilobular region for each of the vectors tested. Semi-quantitative scoring of eGFP staining was performed for peripheral tissues with the liver of an animal treated with scAAVHSC17-CBA-eGFP as an example shown in S3 Fig. Livers from animals treated with either scAAVHSC17-CBA-eGFP or scAAVHSC15-CBA-eGFP had 1+ to 3+ cytoplasmic signal in virtually all hepatocytes. Periportal macrophages had 1+ to 2+ cytoplasmic signal and endothelial cells of the larger blood vessels had a 1+ cytoplasmic signal. eGFP expression in the liver of the animal treated with scAAVHSC7-CBA-eGFP was intense (Fig 16D and 16H) with 2+ to 3+ cytoplasmic signal in all hepatocytes. Again, periportal macrophages, endothelial cells and smooth muscle cells of some blood vessels, had 1+ to 2+ cytoplasmic signal.

In the heart, eGFP staining was generally similar for all three AAVHSCs with 1+ to 3+ cytoplasmic signal in many of the cardiomyocytes of the ventricular and atrial walls (Fig 16J–16L). Cytoplasmic eGFP staining was observed in skeletal myocytes in all skeletal muscle tissues analyzed including the gastrocnemius (Fig 16N–16P), soleus (Fig 16R–16T), biceps (S4G Fig and S4H Fig) and diaphragm (S4I and S4J Fig). In the esophagus (S4 Fig) most skeletal muscle fibers in the muscular wall had 1+ to 3+ eGFP signal in their cytoplasm. Smooth muscle fibers in both the muscular wall and muscularis mucosa had 1+ cytoplasmic signal. No eGFP staining was seen in the esophageal, biceps or diaphragm skeletal muscle sections treated with a non-immune anti-serum control (S4 Fig). Biceps, diaphragm and esophageal tissues were not collected from animals treated with scAAVHSC17-CBA-eGFP or the vehicle control.

**Fig 17:**
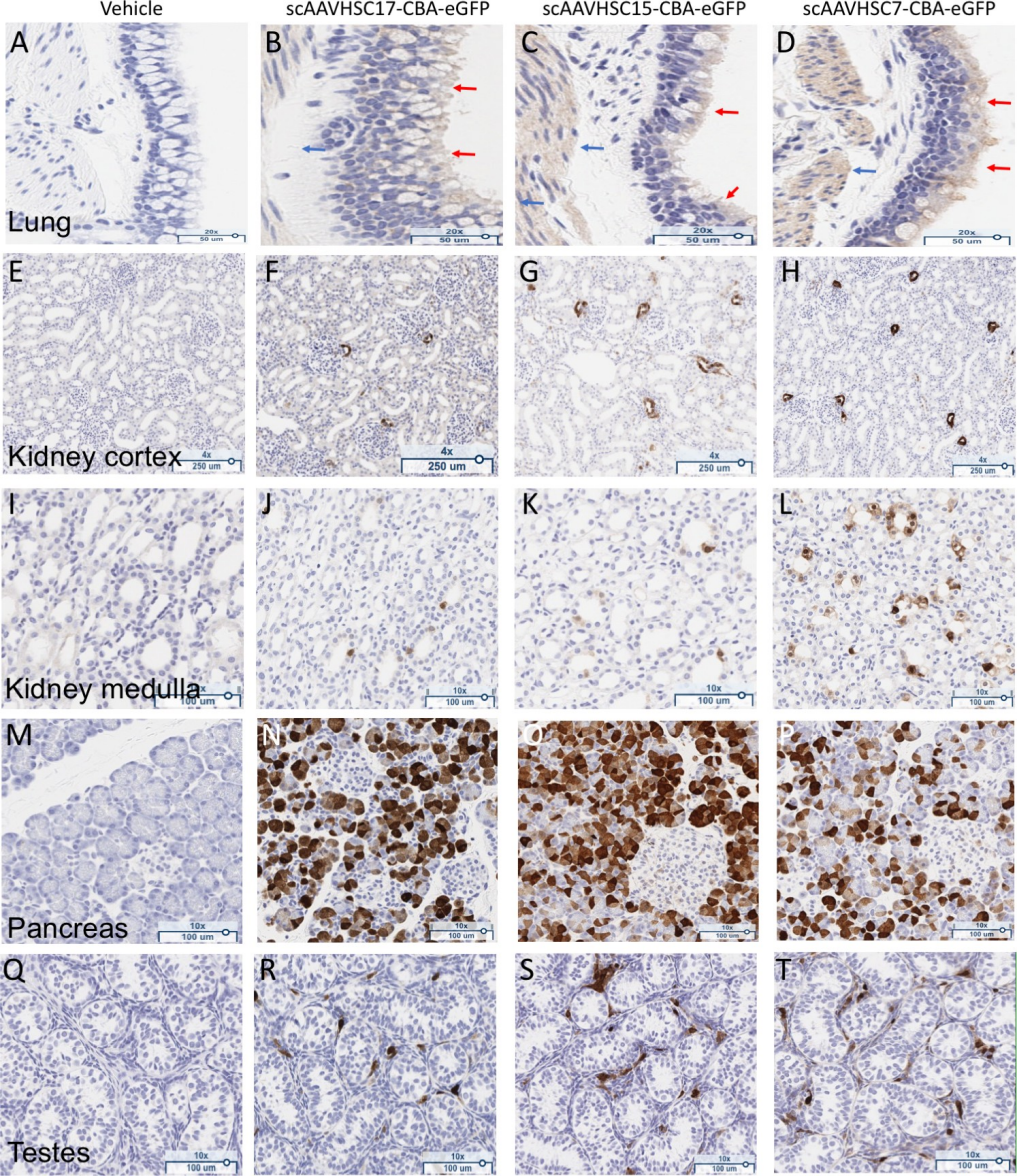
eGFP staining in peripheral tissues of Cynomolgus macaques dosed with scAAVHSC-CBA-eGFP. (A, E, I, M, Q) The animal received an IV dose of vehicle alone. (B, F, J, N, R) The animal received an IV dose of scAAVHSC17-CBA-eGFP. (C, G, K, O, S) The animal received an IV dose of scAAVHSC15-CBA-eGFP. (D, H, L, P, T) The animal received an IV dose of scAAVHSC7-CBA-eGFP. Tissues were processed for eGFP staining as described under Materials and methods. Representative tissues shown are: lung, large airway epithelium (A-D), kidney cortex (E-H); kidney medulla (I-L), pancreas (M-P), and testes (Q-T). Brown staining represents eGFP staining.

Bronchial and bronchiolar epithelium of the lung were positive for eGFP staining after systemic delivery of scAAVHSC-CBA-eGFP (Fig 17B–17D). A modest 1+ to 2+ cytoplasmic signal was evident, particularly in the mucus component of goblet cells, of all treated animals. Lung alveoli were negative for eGFP staining in all treated animals and no eGFP staining was evident within the large airway epithelium of animals treated with vehicle alone (Fig 17A).

eGFP staining in the kidney cortex of treated animals was largely confined to the macula densa or proximal tubular epithelia region directly adjacent to the glomeruli, with 1+ to 3+ cytoplasmic signal observed (Fig 17F–17H). eGFP staining (1+ to 3+ cytoplasmic signal) was also seen in evenly scattered tubular epithelia cells in the medulla (Fig 17J–17L) with relatively greater staining in this region in the animal dosed with scAAVHSC7-CBA-eGFP.

In the pancreas, eGFP staining was largely confined to clusters of acinar cells with 1+ to 3+ cytoplasmic signal observed in each animal treated with scAAVHSC-CBA-eGFP. Islet cells were largely negative for eGFP staining; however, a few scattered cells within some islets were 1+ to 2+ eGFP-positive (Fig 17N–17P).

Spermatogonia were negative for eGFP staining in all treated animals. Individual evenly-spaced Leydig cells had 1+ to 3+ cytoplasmic eGFP signal (Fig 17R–17T) and a few of the epithelial cells of the rete testes had 1+ to 2+ cytoplasmic eGFP signal. Cremaster muscle around the testes showed 1+ to 3+ cytoplasmic eGFP signal.

**Fig 18:**
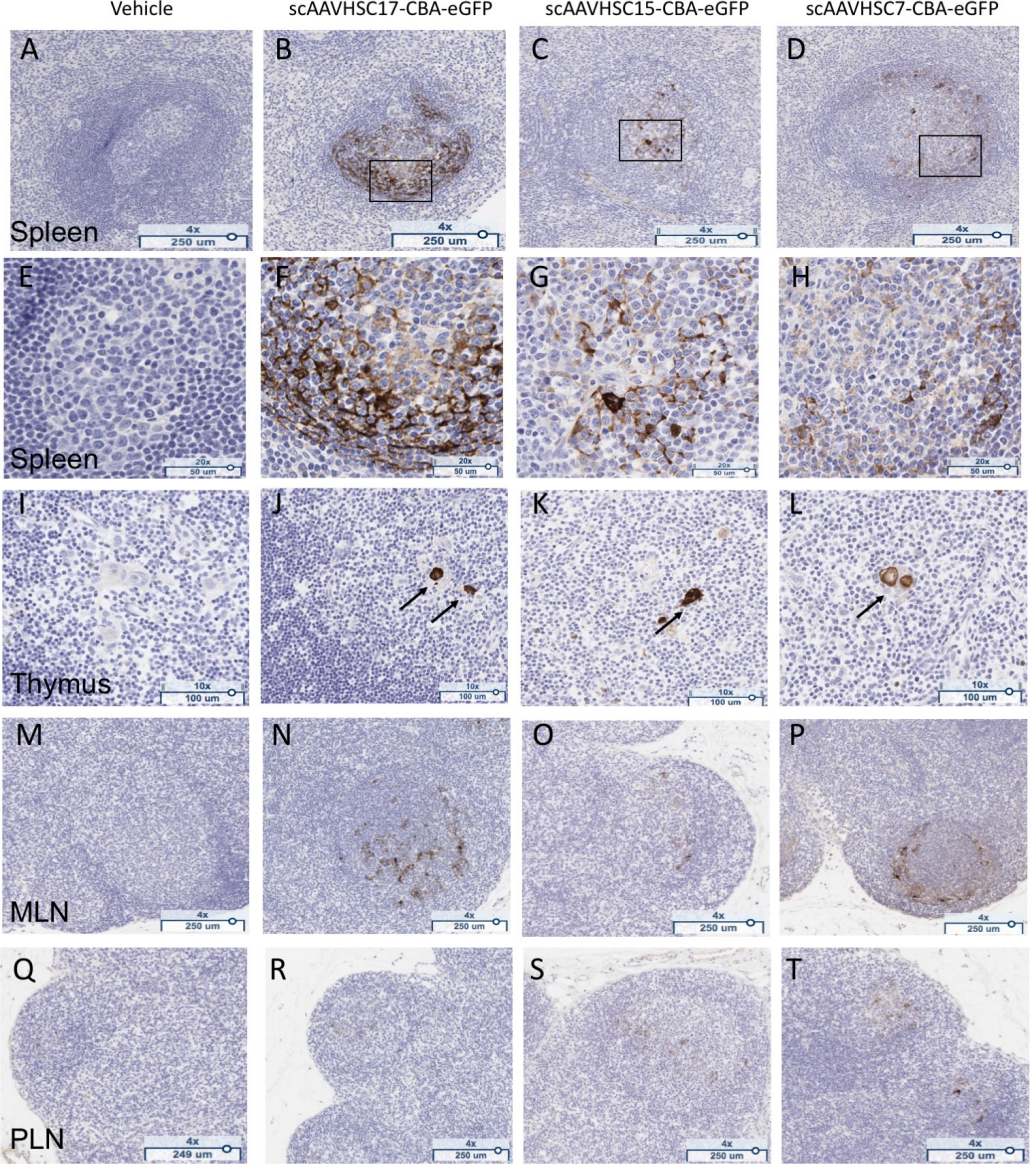
eGFP detection in lymphoid tissue of nonhuman primates treated with scAAVHSC-CBA-eGFP. (A, E, I, M, Q) The animal received an IV dose of vehicle alone. (B, F, J, N, R) The animal received an IV dose of scAAVHSC17-CBA-eGFP. (C, G, K, O, S) The animal received an IV dose of scAAVHSC15-CBA-eGFP. (D, H, L, P, T) The animal received an IV dose of scAAVHSC7-CBA-eGFP. Tissues were isolated two weeks post-dose for eGFP staining as described under Materials and methods. White pulp splenic nodules are shown in A-D with higher magnification views of the boxed areas shown in F-H. eGFP staining in Hassall’s bodies (arrows) of the thymic medulla are shown in I-L, and eGFP staining within the germinal centers of mesenteric (MLN) and peripheral (PLN) lymph nodes are shown in M-P and Q-T, respectively. Brown staining represents eGFP staining. Representative tissues are shown.

Treatment of macaques with IV scAAVHSC17-CBA-eGFP, scAAVHSC15-CBA-eGFP, or scAAVHSC7-CBA-eGFP produced eGFP staining in all immune tissues examined. In the spleen, 1+ to 3+ cytoplasmic eGFP staining was evident in branched and round cells primarily in the mantle zone and germinal centers of most lymphoid follicles (Fig 18B–18D and 18F–18H). The percent of total spleen cells scored as 2+ and 3+ was similar for all three capsids (0.4 to 0.8% eGFP positive). While similar levels of 1+ eGFP staining were seen for animals treated with scAAVHSC7-CBA-eGFP and scAAVHSC15-CBA-eGFP, (0.5% and 0.7% of total spleen cells, respectively), animals treated with scAAVHSC17-CBA-eGFP showed relatively fewer 1+ eGFP stained cells (0.1% of total spleen cells). eGFP staining was also observed within the germinal centers of most follicles of the mesenteric and peripheral lymph nodes in animals treated with scAAVHSC-CBA-eGFP (Fig 18N–18P and 18R–18T). In the thymus, eGFP staining was highly-localized to the epithelial cells of Hassall’s corpuscle, where 1+ to 3+ cytoplasmic signal was observed in animals treated with scAAVHSC-CBA-eGFP (Fig 18J–18L). For animals treated with vehicle alone, no eGFP staining was observed in any of the tissues described above (Fig 17 and Fig 18, far left panels).

Other than a 1+ to 2+ cytoplasmic eGFP-positive signal observed in the entire muscular wall of the duodenum, jejunum and colon only a few scattered epithelial cells, predominately located within the intestinal crypts, showed a 1+ to 3+ cytoplasmic eGFP staining in animals treated with scAAVHSC-CBA-eGFP. The remainder of the intestinal epithelium was largely negative for eGFP staining.

In a sample of skin from the forearm of animals treated with scAAVHSC-CBA-eGFP, 1+ to 2+ cytoplasmic eGFP staining was seen in most arrector pili smooth muscle cells and occasional sweat gland epithelial cells in the dermis. Occasional scattered sebaceous epithelial cells also showed 1+ to 3+ cytoplasmic eGFP staining.

### Biodistribution of eGFP vector genomes in nonhuman primates treated with IV scAAVHSC-CBA-eGFP

**Fig 19:**
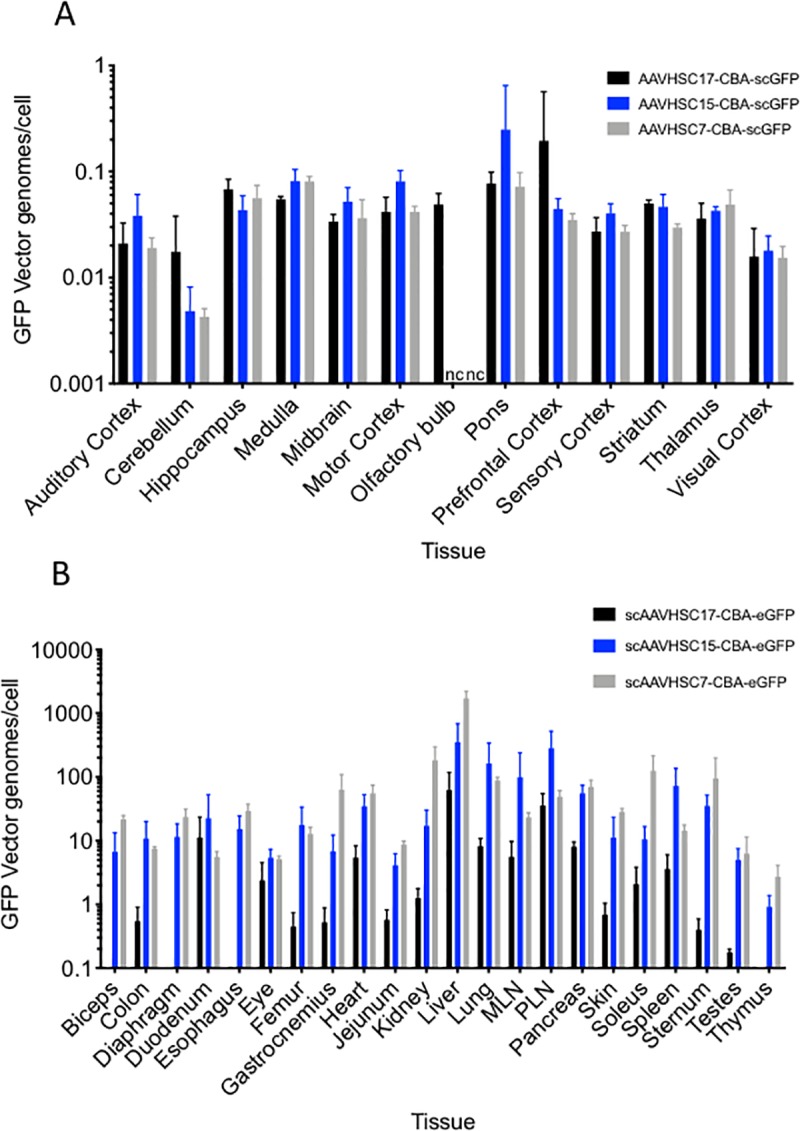
Biodistribution of scAAVHSC-CBA-eGFP in the brain and peripheral organs of nonhuman primates. (A) Levels of vector genomes in the brain of animals treated with an IV dose of scAAVHSC17-CBA-eGFP (black bars), scAAVHSC15-CBA-eGFP (blue bars) or scAAVHSC7-CBA-eGFP (grey bars). (B) Levels of vector genomes within peripheral tissues of animals treated with an IV dose of scAAVHSC17-CBA-eGFP (black bars), scAAVHSC15-CBA-eGFP (blue bars) or scAAVHSC7-CBA-eGFP (grey bars). Two weeks after dosing tissues were harvested and processed for GFP vector genome analyses as described under Materials and methods. The bars in panel A represent the mean ± SD of n = 2–12 pieces of tissue from each brain area of each animal with each assay performed in triplicate. The background level of eGFP vector genomes/cell measured across all brain areas in animals treated with vehicle was 0.009. The bars in panel B represent the mean ± SD of n = 6 tissue sections from each animal treated with scAAVHSC15-CBA-eGFP or scAAVHSC17-CBA-eGFP and n = 3 tissue sections from the animal treated with scAAVHSC7-CBA-eGFP. Eye samples were tissues sections taken through an intact fixed eye from each animal and retinal images are shown. Olfactory bulb samples were only collected from the animals treated with scAAVHSC17-CBA-eGFP and biceps, diaphragm, and esophageal tissues were only collected from animals treated with scAAVHSC15-CBA-eGFP and scAAVHSC7-CBA-eGFP. MLN = mesenteric lymph node, PLN = peripheral lymph node.

Tissue samples were harvested from multiple regions of fixed brain and peripheral tissues from animals treated with scAAVHSC17-CBA-eGFP, scAAVHSC15-CBA-eGFP, or scAAVHSC7-CBA-eGFP for vector genome quantitation. eGFP vector genomes were present in every region of the brain examined with higher levels seen in the pons, hippocampus, medulla and some cortical areas (Fig 19A), findings consistent with the observed eGFP IHC in corresponding regions. The levels of eGFP vector genomes in the cerebellum were low, only animals treated with scAAVHSC17-CBA-eGFP were above those observed with vehicle alone (Fig 19A). In peripheral tissues, the liver contained the highest level of eGFP vector genomes for each of the scAAVHSC-CBA-eGFP tested (Fig 19B). The order of relative potency for transduction of liver, measured by eGFP vector genomes, was scAAVHSC7-CBA-eGFP > scAAVHSC15-CBA-eGFP>>scAAVHSC17-CBA-eGFP, findings consistent with eGFP expression measured by IHC. Immune tissues and lung from animals treated with scAAVHSC-CBA-eGFP also showed relatively high levels of eGFP vector genomes (Fig 19B). For scAAVHSC7-CBA-eGFP, high levels of eGFP vector genomes were also present in kidney, soleus and gastrocnemius muscles and sternum (Fig 19B); for scAAVHSC15-CBA-eGFP high levels of eGFP vector genomes were also detected within pancreas, heart and sternum (Fig 19B); and for scAAVHSC17-CBA-eGFP duodenum, pancreas and heart also showed high levels of eGFP vector genomes (Fig 19B). In the duodenum, the eGFP vector genomes were likely due to transduction of contaminating skeletal/smooth muscle within the isolated tissues. In most of these tissues, transduction by scAAVHSC7-CBA-eGFP and scAAVHSC15-CBA-scGFP exceeded that of scAAVHSC17-CBA-scGFP (Fig 19B).

## Discussion

The results of this study show that three Clade F AAVs, AAVHSC17, AAVHSC15 and AAVHSC7, showed widespread distribution in the nervous system and multiple peripheral tissues following IV delivery in Cynomolgus macaques. eGFP vector genome and eGFP IHC analyses showed transduction and transgene expression throughout the brain in both white and grey matter regions at two weeks post-dose, with the highest levels seen in the pons and LGN for each of the AAVHSCs tested. Detection of eGFP staining was also seen throughout the cortex and midbrain structures predominately within glial cells. While an enrichment in glial transduction was observed in most of these cerebral regions, eGFP staining was also confirmed within neuronal cell bodies, proximal dendritic arborization, and axons throughout. These data are consistent with previous reports for another Clade F virus, AAV9, which can effectively cross the BBB after IV administration in nonhuman primates [18, 19] supporting the claim that the ability to efficiently cross the BBB may be a feature of Clade F AAVs. A number of native and engineered recombinant AAVs from other clades have been shown to cross the BBB in mice following systemic delivery [25–28] for reviews and [29–32] with only a few of these vectors studied in larger animals to date [33]. In some cases moreover, translation of the biodistribution data observed in mice to nonhuman primates has been poor [21, 34, 35], emphasizing the importance of studying the tissue tropism of AAV in large animals that may more closely reflect the underlying biology in humans.

In addition to transduction of the brain, AAVHSCs effectively targeted the spinal cord and associated DRG demonstrating tropism to the broader nervous system, in glial and neuronal profiles and the ability to cross the BNB in addition to the BBB. Of note, the neuron to glia transduction was reversed to that of the LGN in the DRG. Transduction of neurons with the sensory ganglia has been observed for Clade F AAV in other studies in nonhuman primates [18] consistent with the data for the AAVHSCs in the current report. The mechanism for enhanced sensory neuron tropism in the periphery is not yet clear. In the spinal cord gray matter, astrocyte-like profiles and large motor neurons showed extensive eGFP staining. For each scAAVHSC-CBA-eGFP tested, eGFP staining was highest within the lumbar region with varying immunoreactivity patterns across the rostro-caudal gradient of the spinal cord. The reason for the relative lack of eGFP staining within the thoracic region is not clear, possibly signifying differences in vascular or regional expression of AAV binding receptors. The absence of large pools of motor neurons within this region may also give an appearance of lower signal since they predominate in the regions innervating the limbs (to this note, the brachial enlargement innervating the forelimbs was not evaluated). The extensive eGFP staining in sensory neurons of the DRG and in the proximal axons of the spinal nerve and the dorsal roots seen with systemic delivery of AAVHSCs extend the findings observed with AAV9 [18, 19] to other members of AAV Clade F.

The mechanism by which Clade F AAVs traverse the BNB or BBB after systemic delivery is not yet established. In a cell culture BBB model system, using brain microvascular endothelial cells (BMVECs), AAV9 appeared to cross the endothelial barrier by transcytosis through tubule-like structures with little or no transduction of the BMVECs or disruption of the BMVEC barrier [36]. Primary human astrocytes, on the other hand, were readily transduced following passage of AAV9 through the endothelial barrier. AAV2, in contrast, was endocytosed resulting in transduction of the BMVECs [36] with little or no transduction of underlying astrocytes [36]. These AAVs thus appear to follow divergent pathways through a brain-derived endothelial barrier *in vitro* suggesting a mechanism for the BBB permeability to Clade F AAV *in vivo*. These data are consistent with our findings in the present study where the vascular endothelium in nonhuman primate brain was largely devoid of eGFP staining following IV delivery of scAAVHSC17-, scAAVHSC15-, or scAAVHSC7-CBA-eGFP. AAV9 appears to utilize glycans with terminal N-linked galactose as its primary cellular receptor in conjunction with the laminin receptor as co-receptor [37, 38]. Whether the distribution of these molecules, the AAVR [39] or other pathways [40, 41] on the brain endothelium render the BBB permeable to Clade F AAV is not yet understood. Enhanced BBB crossing by the modified AAV9 vectors, AAV-PHP.B and AAV-PHP.eB, [34] is likely mediated by the GPI-anchored protein LY6A, also known as SCA-1, in C57BL/6J mice [42]. While these findings appear to be strain-specific and do not translate to nonhuman primates [21, 35], lipid raft-associated GPI-anchors could play a key role in AAV transcytosis and transduction as shown for a number of other viruses [43]. Whether a primate ortholog of LY6A or another GPI-anchored membrane protein mediates the transport of Clade F AAVs across the BBB remains to be determined.

As noted above, eGFP staining in the brain was predominately localized in glial cells which on thionine counterstaining exhibited the morphology of astrocytes. This observation was confirmed by colocalization of eGFP with classic glial cell markers. While astrocytic-like staining was most abundant, transduction of other glial cell types in the CNS such as oligodendrocytes, cells of the choroid plexus and Müller glia or the PNS such as satellite cells were also observed suggesting a potential for transduction by these capsids of a large and diversified number of glial cell type in the nervous system. In a similar manner, eGFP-positive neuronal profiles were observed throughout the nervous system and their identity confirmed by co-labeling with neuronal markers.

In the periphery, AAVHSCs exhibit high levels of tropism for the liver and skeletal muscle throughout the body. The eGFP staining and vector genome data suggest that all three exhibit high tropism for the liver in addition to effectively transducing multiple other tissues, including skeletal muscle and heart, after a single IV injection. In addition to the work described here, we have observed differences in transgene biodistribution after a single IV injection of these and additional AAVHSCs in mice suggesting that some vectors may have greater tropism for targeting specific tissues [44]. It should be noted that eGFP staining by IHC was generally correlated with eGFP vector genomes in corresponding tissue. This was especially true for relatively homogenous tissues such as liver. However, the high muscle tropism of the AAVHSCs complicates interpretation of the vector genome analyses where contaminating or integral muscle tissue may be present (as was observed in the duodenal sample of the animals treated with scAAVHSC17-CBA-eGFP). The multinucleated nature of each myofiber also complicates the vector genome analyses as a myofiber can be strongly eGFP positive yet have few vector genomes per nucleus. Nevertheless, the widespread transduction of multiple peripheral tissues is expected with systemic administration of AAVs. Our data agrees with published work for systemic delivery of the other Clade F member; AAV9 [18, 19], where these tissues are also targeted. Moreover, the differences observed in regional and cell type tropism with the characterized AAVHSCs allows for tuning of capsid selection for diseases of the nervous system. By overlaying the key ascending and descending neuronal pathways connecting the periphery to the CNS (and vice versa) and/or the cell-types mostly affected in a given neurological disease (glia vs. neurons or both), one can select the biologically relevant capsids for the desired neuronal/glial endpoints and also the peripheral organs that may benefit from the therapy via an IV route of administration.

A recent report documented acute hepatotoxicity, coagulopathy, and severe inflammation in a rhesus macaque and toxicity to sensory neurons within the DRG in newborn piglets that received an IV dose (2 x10^14^ vg/kg) of an engineered AAV9 variant AAVhu68 packaging a human SMN transgene [45]. We have not observed these findings following systemic dosing of normal 4- to 5-month old cynomolgus macaques with scAAVHSC-CBA-eGFP at the doses tested and over the time course utilized. All animals appeared healthy in our study with no behavioral changes noted and livers appeared normal at necropsy. Our data are consistent with gene therapy trials in patients with spinal muscular atrophy treated with systemic AAV9-*SMN1* at 2 x10^14^ vg/kg that have shown remarkable efficacy with little observed toxicity [5]. Zolgensma was, in fact, recently approved by the FDA for pediatric SMA patients at a IV dose of 1.1 x10^14^ vg/kg [20].

The broad PNS/CNS distribution of the naturally-occurring Clade F AAVHSCs, suggests their potential for treating neurological diseases by systemic administration. The success of IV AAV9-*SMN* in treating patients with spinal muscular atrophy supports and validates this view. The hepatic and peripheral tissue tropism further suggests an AAVHSC-mediated approach for treating genetic diseases of the periphery including liver, skeletal muscle and heart. A factor that can limit the therapeutic utility of systemically delivered AAVs is the level of anti-AAV neutralizing antibodies present within the circulation [46]. We have recently shown that the seroprevalences of neutralizing antibodies targeting AAVHSC17 and AAVHSC15 in humans are low [47] enabling the use of these AAVHSCs for systemic delivery within the general population. The AAVHSCs have shown high-efficiency, nuclease-free gene editing both *in vitro* with cultured human and rodent cells and *in vivo* in mice [11]. An approach coupling the wide tissue tropism of the AAVHSCs following IV delivery with homology-driven gene editing or gene transfer using tissue-specific regulatory elements may have therapeutic potential for durably treating a wide variety of genetic diseases in humans.

## Supporting information

S1 Fig High magnification photomicrographs of eGFP staining within glial and neuronal cells in multiple brain regions of nonhuman primates treated with an IV dose of scAAVHSC-CBA-eGFP. (A and D) Macaques received either scAAVHSC17-CBA-eGFP, (B and E) scAAVHSC15-CBA-eGFP or (C and F) scAAVHSC7-CBA-eGFP. eGFP staining within cortical glia (A and F, red arrows = glial cells), neurons in putamen (B, black arrow = axon; blue arrows = dendrites; and D, blue arrows = dendrites), and cerebellar Purkinje cells (C, black arrow = axon; blue arrows = dendrites) and Bergmann glial cells (E, red arrows). Each scale bar in A, B, E, and F represents 50 μm. The scale bars in C and D represent 100 μm.
(TIF)

S2 Fig Endothelial cells of the brain vasculature show little or no eGFP staining following IV delivery of scAAVHSC17-CBA-eGFP. Animals were treated with (A, B) scAAVHSC17-CBA-eGFP or (C, D) vehicle alone and brain tissues were harvested and processed for eGFP staining as described under Materials and methods. A and C: cortex; B and D, pons. Asterisks show brain blood vessels, large blue arrows show glial eGFP staining, and small black arrows show neuronal eGFP staining. The scale bars in A and C represent 50 μm and the scale bars in B and D represent 25 μm.
(TIF)

S3 Fig Semi-quantitative scoring of eGFP staining in peripheral tissues. Example shown is a liver section from a nonhuman primate treated with scAAVHSC17-CBA-eGFP. eGFP-positive cells were scored with increasing staining intensity as 1+, 2+ or 3+ in a blinded manner by a board-certified veterinary histopathologist at Charter Preclinical Services.
(TIF)

S4 Fig eGFP staining within skeletal muscle of nonhuman primates following IV dosing with scAAVHSC-CBA-eGFP. (A, B, D, E, G, and I) Animals were treated with either scAAVHSC15-CBA-eGFP or (C, F, H, J, K, L) scAAVHSC7-CBA-eGFP and tissues were isolated and processed for eGFP staining as described under Materials and methods. Samples in A, C, D, F, and G-J were stained with an anti-eGFP antibody and samples in B, E, K, and L were stained with an equivalent concentration of a non-immune sera. (A-F) Esophageal tissues. (G, H, and K) bicep tissues. (I, J, and L) diaphragm tissues. Higher magnification views of the boxed areas in A-C are shown in D-F, respectively. The tissues shown in this figure were not collected from animals treated with scAAVHSC17-CBA-eGFP. Brown staining represents eGFP staining in representative tissues.
(TIF)
