# Supplementary figures and images for: Clade F AAVHSCs cross the blood brain barrier and transduce the central nervous system in addition to peripheral tissues following intravenous administration in nonhuman primates
Source: PLoS One. 2019 Nov 26;14(11):e0225582. doi: 10.1371/journal.pone.0225582 (PMC6879147; doi:10.1371/journal.pone.0225582)

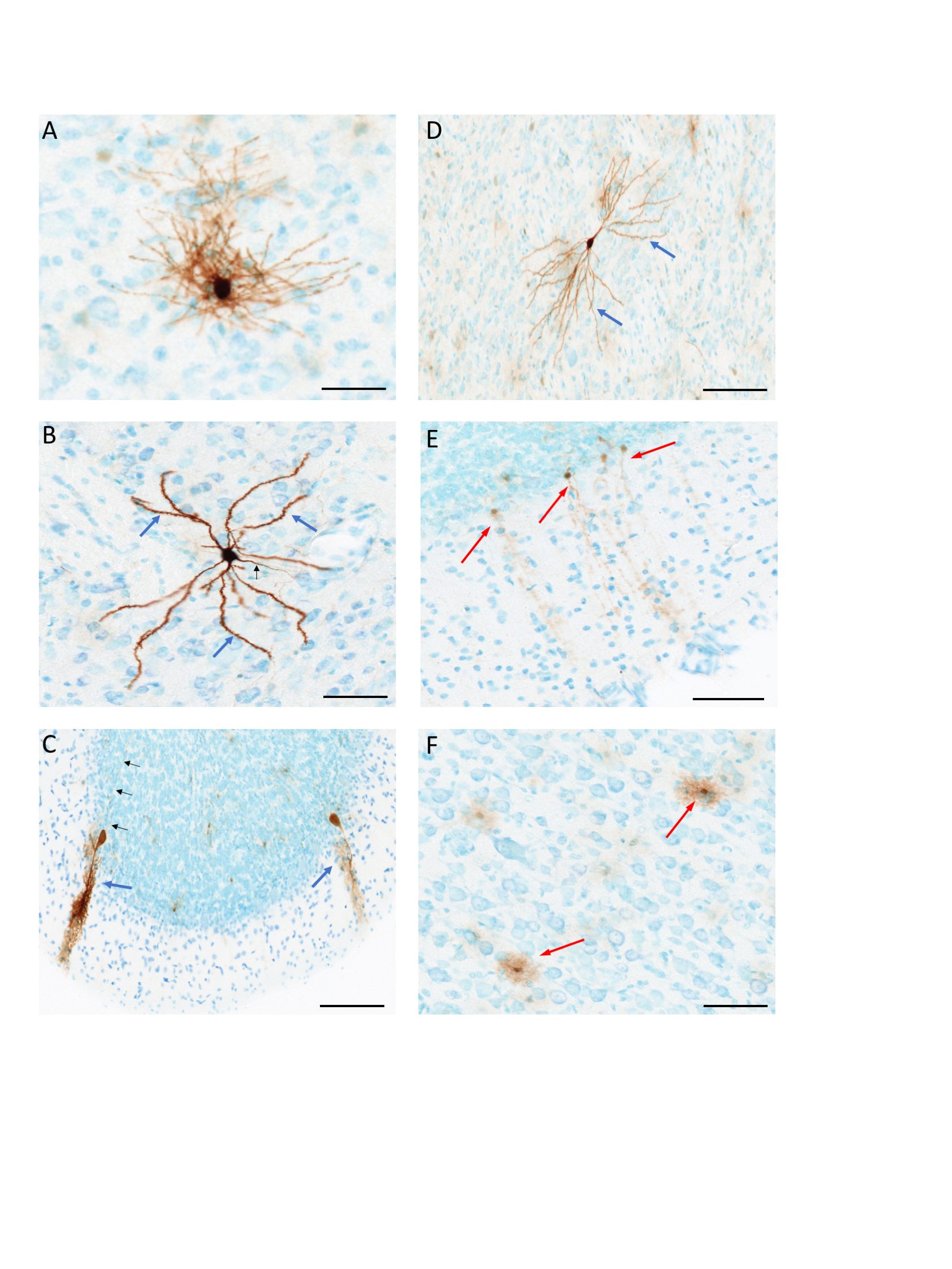

Supplement: S1 Fig — (A and D) Macaques received either scAAVHSC17-CBA-eGFP, (B and E) scAAVHSC15-CBA-eGFP or (C and F) scAAVHSC7-CBA-eGFP. eGFP staining within cortical glia (A and F, red arrows = glial cells), neurons in putamen (B, black arrow = axon; blue arrows = dendrites; and D, blue arrows = dendrites), and cerebellar Purkinje cells (C, black arrow = axon; blue arrows = dendrites) and Bergmann glial cells (E, red arrows). Each scale bar in A, B, E, and F represents 50 μm. The scale bars in C and D represent 100 μm. (TIF) [file pone.0225582.s001.tif]

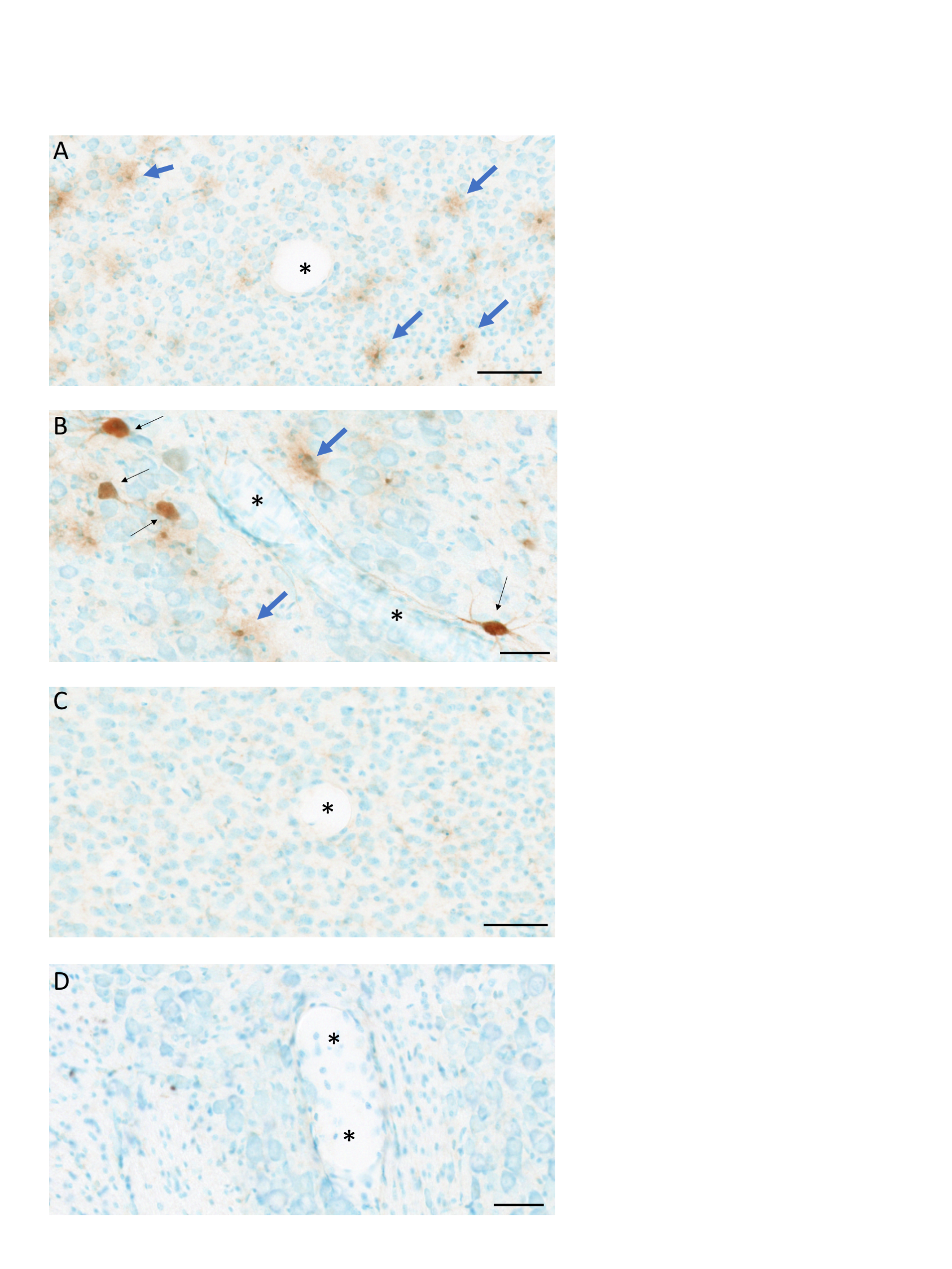

Supplement: S2 Fig — Animals were treated with (A, B) scAAVHSC17-CBA-eGFP or (C, D) vehicle alone and brain tissues were harvested and processed for eGFP staining as described under Materials and methods. A and C: cortex; B and D, pons. Asterisks show brain blood vessels, large blue arrows show glial eGFP staining, and small black arrows show neuronal eGFP staining. The scale bars in A and C represent 50 μm and the scale bars in B and D represent 25 μm. (TIF) [file pone.0225582.s002.tif]

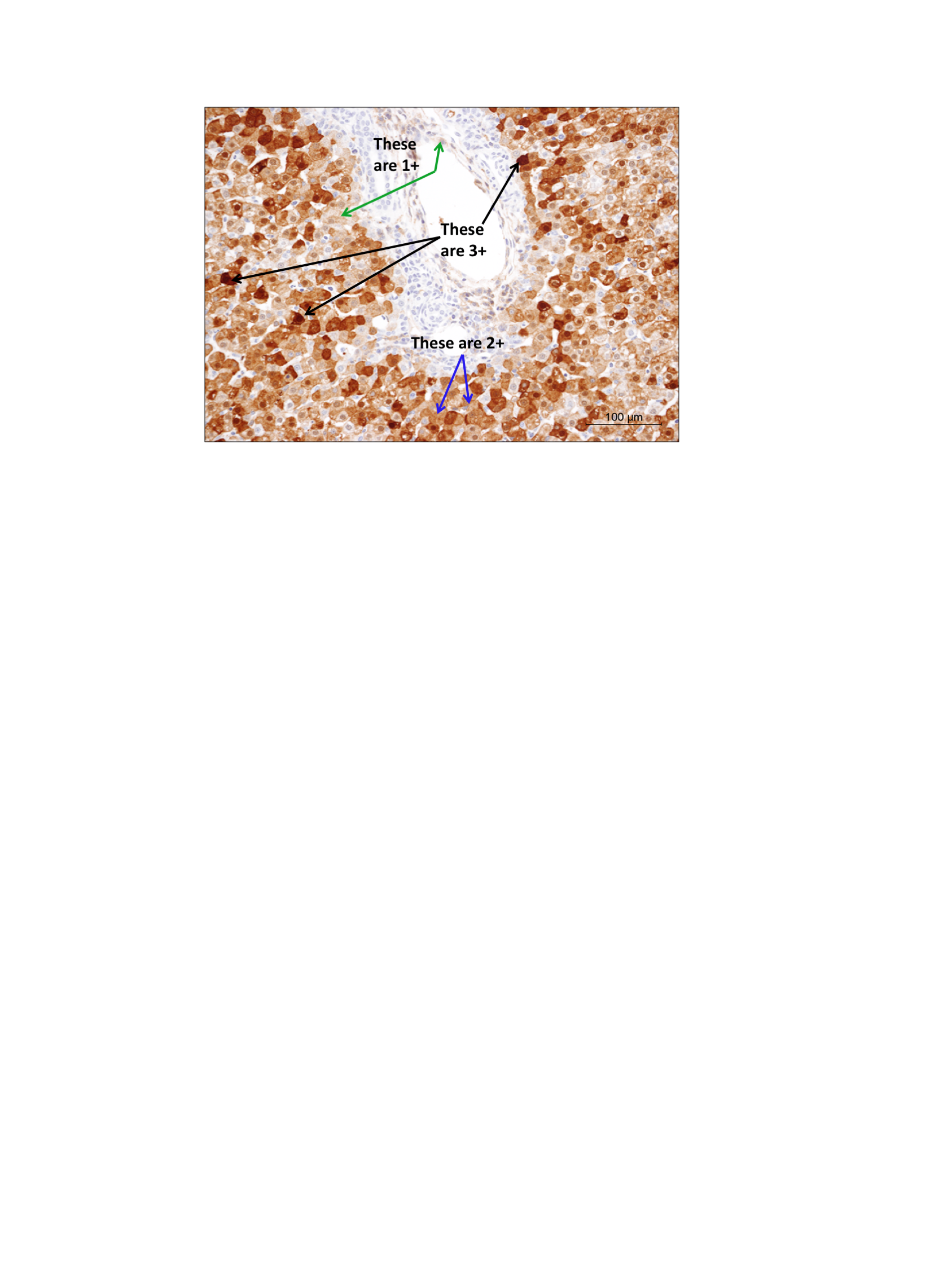

Supplement: S3 Fig — Example shown is a liver section from a nonhuman primate treated with scAAVHSC17-CBA-eGFP. eGFP-positive cells were scored with increasing staining intensity as 1+, 2+ or 3+ in a blinded manner by a board-certified veterinary histopathologist at Charter Preclinical Services. (TIF) [file pone.0225582.s003.tif]

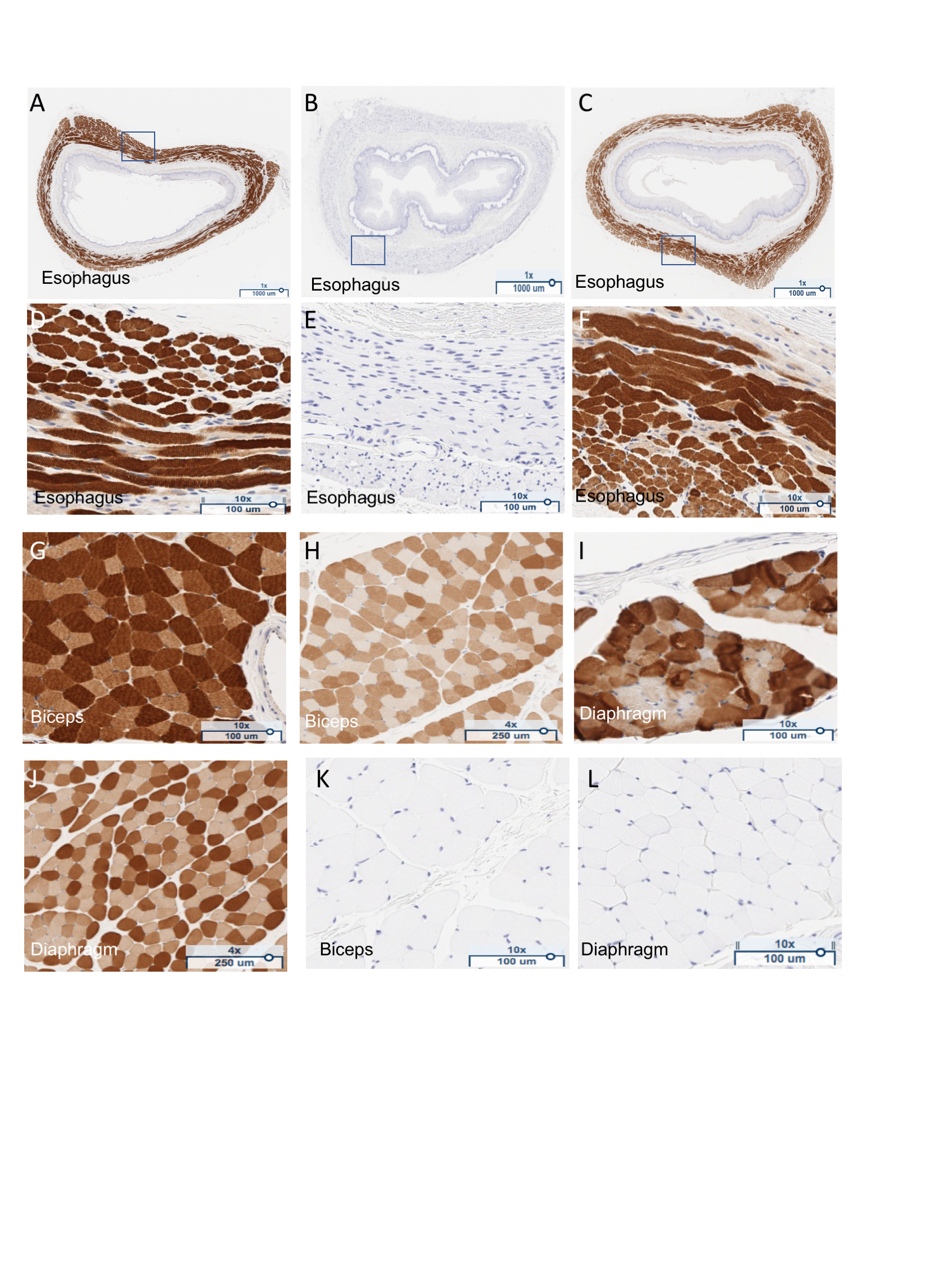

Supplement: S4 Fig — (A, B, D, E, G, and I) Animals were treated with either scAAVHSC15-CBA-eGFP or (C, F, H, J, K, L) scAAVHSC7-CBA-eGFP and tissues were isolated and processed for eGFP staining as described under Materials and methods. Samples in A, C, D, F, and G-J were stained with an anti-eGFP antibody and samples in B, E, K, and L were stained with an equivalent concentration of a non-immune sera. (A-F) Esophageal tissues. (G, H, and K) bicep tissues. (I, J, and L) diaphragm tissues. Higher magnification views of the boxed areas in A-C are shown in D-F, respectively. The tissues shown in this figure were not collected from animals treated with scAAVHSC17-CBA-eGFP. Brown staining represents eGFP staining in representative tissues. (TIF) [file pone.0225582.s004.tif]
